# *Muribaculum intestinale* restricts *Salmonella* Typhimurium colonization by converting succinate to propionate

**DOI:** 10.1093/ismejo/wraf069

**Published:** 2025-04-18

**Authors:** Zhenyu Wang, Shuaishuai Kang, Zhenhua Wu, Xiaoyi Liu, Xiangyu Zhang, Yujun Wu, Yang Wen, Xingjian Zhou, Guolong Zhang, Junjun Wang, Dandan Han

**Affiliations:** State Key Laboratory of Animal Nutrition and Feeding, College of Animal Science and Technology, China Agricultural University, Beijing 100193, China; State Key Laboratory of Animal Nutrition and Feeding, College of Animal Science and Technology, China Agricultural University, Beijing 100193, China; State Key Laboratory of Animal Nutrition and Feeding, College of Animal Science and Technology, China Agricultural University, Beijing 100193, China; State Key Laboratory of Animal Nutrition and Feeding, College of Animal Science and Technology, China Agricultural University, Beijing 100193, China; State Key Laboratory of Animal Nutrition and Feeding, College of Animal Science and Technology, China Agricultural University, Beijing 100193, China; State Key Laboratory of Animal Nutrition and Feeding, College of Animal Science and Technology, China Agricultural University, Beijing 100193, China; State Key Laboratory of Animal Nutrition and Feeding, College of Animal Science and Technology, China Agricultural University, Beijing 100193, China; State Key Laboratory of Animal Nutrition and Feeding, College of Animal Science and Technology, China Agricultural University, Beijing 100193, China; Department of Animal and Food Sciences, Oklahoma State University, Stillwater, OK 74078, USA; State Key Laboratory of Animal Nutrition and Feeding, College of Animal Science and Technology, China Agricultural University, Beijing 100193, China; State Key Laboratory of Animal Nutrition and Feeding, College of Animal Science and Technology, China Agricultural University, Beijing 100193, China

**Keywords:** dietary fiber, gut microbiota, colonization resistance, *Muribaculum intestinale*, succinate

## Abstract

Insufficient dietary fiber intake is associated with dysbiosis and compromised colonization resistance (CR) to enteric infections. However, a detailed understanding of the relationship between dietary fiber insufficiency and CR remains elusive. Our study aimed to delineate the impact of fiber deprivation on gut microbiome and CR in a murine model with *Salmonella* Typhimurium infection. Our findings indicate that dietary fiber deprivation resulted in impaired CR and depletion of commensal bacteria *Muribaculaceae*. By combining dietary switch, FMT, and genomic analysis, we identify *Muribaculum intestinale* as a candidate bacterium, capable of converting succinate into propionate. Oral administration of *Muribaculum intestinale* augmented CR to *Salmonella* Typhimurium, accompanied by succinate reduction and propionate elevation. Dietary supplementation of propionate, but not succinate, enhanced CR to *Salmonella* Typhimurium in mice consuming a fiber-free diet. Taken together, our research identified a crucial metabolic pathway encoded by gut microbiome underlying CR, providing an intervention strategy for combatting enteric infections among Western diet-consuming populations.

## Introduction

The mammalian intestinal tract harbors trillions of microbes, including bacteria, viruses, archaea, and fungi. This intricate and dense microbial community has forged a mutualistic relationship with the host over millions of years of co-evolution [1]. Commensal bacteria are known to be capable of limiting the infections of pathogens through a mechanism known as colonization resistance (CR) [2]. Microbiota-mediated CR against infections can be.

categorized into direct and indirect mechanisms [3]. Major direct mechanisms of CR encompass nutrient competition, spatial occupancy, contact-dependent killing, and production of inhibitory molecules such as short-chain fatty acids (SCFAs), secondary bile acids, and bacteriocins [4–7]. Indirectly, intestinal microbiota mediates CR by maintaining a hypoxic environment, promoting intestinal and epithelial barrier integrity, as well as eliciting host innate and adaptive immune responses [8–10]. However, there remains a significant gap in our current understanding of the CR mechanisms.

Dietary fiber intake plays a crucial role in influencing CR against enteric pathogens [11]. The fermentation of dietary fiber by commensal bacteria in the large intestine, such as the cecum and colon, results in the production of a high concentration of SCFAs, creating a highly anoxic environment that inhibits the invasion and colonization of enteric pathogens [12]. The Western diet typically provides a daily dietary fiber intake less than the recommended 28–35 g, leading to dysbiosis [13,14]. In a humanized mouse model inhabited by 14 bacterial strains, dietary fiber deprivation abolished fiber-degrading bacteria, showing reduced SCFA production and increased *Citrobacter rodentium* colonization [15]. In conventional mice, the Western diet also promoted the proliferation of *Salmonella* Typhimurium in a fat-dependent manner [11]. However, the specific causal relationship between dietary fiber deprivation, gut microbiome, metabolites, and pathogen susceptibility remains largely unexplored.

This study aimed to elucidate the mechanism by which dietary fiber deprivation adversely affects CR in a *S.* Typhimurium murine infection model. We demonstrated that dietary fiber deprivation causes dysbiosis and compromises CR against *S.* Typhimurium. Through a combination of dietary reversal, fecal microbiota transplantation (FMT), and targeted metabolomics, we identified succinate and *Muribaculum intestinale* as key mechanisms mediating CR against *S.* Typhimurium. Our findings confirmed that *M. intestinale* confers resistance to *S.* Typhimurium colonization by converting succinate into propionate.

## Material and methods

### Animals and diets

All animal protocols were approved by China Agricultural University Animal Care and Use Committee (Beijing, China). For all experiments, 6- to 8-week-old male C57BL/6 J mice were used and purchased from Sipeifu Biotechnology (Beijing, China). All mice were bred and maintained under specific pathogen-free (SPF) conditions in filter-top cages in the mouse facility of China Agricultural University. After a week of acclamation, mice were fed either a standard chow (ST, Sipeifu Biotech) or a fiber-free (FF) chow as described elsewhere (Table S1) [15]. Sterile water and diet were provided *ad libitum* throughout the experiments.

### Bacterial strains

A streptomycin-resistant strain of *S. enterica* serovar Typhimurium SL1344 (*S.* Typhimurium) used in this study was purchased from the American Type Culture Collection (ATCC). *S.* Typhimurium was grown on LB agar plates supplemented with 50 mg/L streptomycin overnight at 37°C. One colony was re-suspended in 10 ml LB broth and grown overnight at 37°C with shaking at 200 rpm. Overnight bacterial culture (0.5 ml) was inoculated in 10 ml LB broth and further grown for another 6–8 h, followed by centrifugation at 9,000 × *g* for 5 min and re-suspension in ice-old sterile PBS for inoculation. *Muribaculum intestinale* YL27 (DSM 28989) was purchased from DSMZ (Braunschweig, Germany) and grown in a modified version of PYG medium (DSMZ medium 104c) for 48 h at 37°C under anoxic conditions without shaking.

### Animal infection

For infections, each mouse was administrated with 5 × 10 [7] CFU of *S.* Typhimurium in 200 μl PBS or mock-infected with 200 μl sterile PBS. Fresh fecal pellets were collected 4 days post infection (dpi). Additionally, all mice were sacrificed at 4 dpi, and the cecal and colonic contents, mesenteric lymph nodes, spleens, and livers were collected, homogenized in nine volumes of sterile PBS in TissueLyser (Qiagen), and serially diluted [10-1–10-3], with 100 μl of each dilution plated on MacConkey agar (Oxiod, CM0007) containing 50 mg/L streptomycin. The detection limit is 10 CFU/g for the feces, digesta, and tissues.

### FMT

Fresh fecal pellets were collected from donor mice, stored on ice, homogenized in nine volumes of sterile PBS, and gavaged immediately into 12 C57BL/6 J recipient mice (100 μl per recipient) housed in individual cages under the SPF condition. The FMT procedure was performed daily for 14 days.

### 16S rRNA gene amplicon sequencing

DNA extraction and 16S rRNA gene sequencing were performed as described previously [16]. In brief, microbial DNA was extracted from the intestinal luminal content samples using FastDNA SPIN Kit for soil (MP Biomedicals, USA). Extracted DNA was quantified using NanoDrop 2000 (Thermo Fisher Scientific, USA). DNA quality was evaluated by agarose gel electrophoresis. The V3-V4 region of the bacteria 16S rRNA gene was amplified with primer pairs 338F (5’-ACTCCTACGGGAGGCAGCAG-3′) and 806R (5’-GGACTACHVGGGTWTCTAAT-3′) in an ABI GeneAmp 9700 PCR thermocycler (ABI, CA, USA) [16]. PCR products were purified, quantified, pooled, and sequenced on the MiSeq PE300 platform (San Diego, USA) according to the standard protocols of Majorbio Bio-Pharm Technology (Shanghai, China).

Raw sequencing reads were demultiplexed and processed using QIIME 2 (version 2020.2) [17]. Quality control and denoising were performed using DADA2 with default parameters to generate amplicon sequence variants (ASVs) [18]. To avoid the bias resulting from variable sequencing depth, all samples were rarefied to the minimal sequence depth. ASVs were taxonomically classified using scikit-learn and the SILVA 138 database [19,20]. Both α-and β-diversities were calculated using vegan package (version 2.5–6) in R software. Principal coordinate analysis (PCoA) was performed using weighted Bray-Curtis distance metrics. Statistics were performed using PERMANOVA with the adonis2 function of “vegan” package (999 permutations). Differential enrichment of bacterial taxa was identified using “mp_diff_analysis” embedded in the MicrobiotaProcess package [21].

### Metagenomics sequencing and analysis

Microbial genomic DNA was extracted using the E.Z.N.A Soil DNA Kit (Omega Bio-tec, USA). The concentration and purity of each sample were determined with TBS-380 (Promega, USA) and NanoDrop 2000 (Thermo Fisher Scientific, USA), respectively. The quality of extracted DNA was checked on 1% agarose gel. DNA was fragmented to ~400 bp using Covaris M220 (Gene Company, China). Adapter ligation, cleanup, and enrichment were performed using NEXTFLEX Rapid DNA-Seq Kit (Bioo Scientific, USA). Shotgun metagenomic sequencing was performed on a NovaSeq System (Illumina) by Novogene (Beijing, China). Quality control was performed using fastp (version 0.19.4) with parameter “--cut_by_quality3 -W 4 -M 20 -n 5 -c -l 50 -w 3” [22]. Kneaddata (version 2.4.1) was used to remove reads aligned to the swine genome [23]. Microbial taxonomic profiles were generated using MetaPhlAn4 (version 3.0.7) [24]. Differentially enriched species were identified using “mp_diff_analysis” embedded in the MicrobiotaProcess package [21].

### Recovery of bacteria genomes from metagenomics data

Spades (version 3.15.22) was employed to assemble the metagenomics reads of each sample using “meta” mode and default parameters [25]. Contigs shorter than 1 kb were removed before binning. Genome reconstruction from metagenomics sequences was performed using metaWRAP [26]. Metagenome-assembled genomes (MAGs) were constructed from contigs using default parameters and two binning algorithms of “—maxbin2” and “-metabat2”. Bins were further refined using the metaWRAP refinement module. CheckM (version 1.1.13) was used to evaluate the completeness and contamination of MAGs [27]. MAGs with completeness <50% and contamination >5% (low-quality genome) were filtered out. The resulting MAGs were dereplicated using dRep (version 2.2.3) with parameter “-nc 0.30 -pa 0.9 -sa 0.95” to obtain species-level clusters [28]. Briefly, MAGs were divided into primary clusters using Mash at 90% average nucleotide Identity (ANI). Each primary cluster was then used to form secondary clusters at the threshold of 95% ANI with at least 30% overlap between genomes. A total of 138 MAGs were retained after quality control and dereplication. CheckM was performed to check the quality of dereplicated MAGs [27], which were further subjected to GTDB-tk (version 1.5.1) for taxonomy classification [29,30].

### Annotation of potential metabolic pathways

The reconstructed *M. intestinale* genome was annotated using eggNOG mapper against the eggNOG database (version 5.0) [31,32]. The KEGG orthology (KO) list was extracted and subjected to clusterprofiler for enrichment analysis using the *M. intestinale* reference genome as reference. GutSMASH was also used to predict the metabolic potential of the reconstructed *M. intestinale* genome and the reference genome of *M. intestinale* downloaded from the NCBI Refseq database [33].

### Metabolomics

The colon contents were thawed on ice, and ~5 mg were transferred to 25 μl water for homogenization. After mixed with methanol, the homogenates were processed for targeted metabolomics by Metabo-Profile Biotechnology (Shanghai, China). Raw data files were processed using MassLynx software (v4.1, Waters, Milford, MA, USA) for peak integration, calibration, and quantification of each metabolite.

### Quantification of Lcn2 and IFN-γ by ELISA

Lipocalin-2 (Lcn2) concentration in fecal samples as determined with a Mouse Lipocalin-2/NGAL DuoSet ELISA kit (R&D Systems, Bio-Techne, MN, USA) according to manufacturer’s protocol. The concentrations of interferon (IFN)-γ in the spleen were analyzed with a Mouse IFN-gamma DuoSet ELISA kit (R&D Systems, Minneapolis, MN, USA) according to manufacturer’s protocol.

### Metabolite measurement

SCFA and succinate concentrations were measured in the fecal samples as described [16]. Briefly, samples were thawed on ice, and ~0.5 g was added to 8 ml deionized water for homogenization. After centrifugation at 13 000 × *g* for 5 min, the supernatant was diluted 50-fold and filtered through a 0.22 μm filter (Millipore, Bedford, OH). High-performance ion chromatography was performed with 25 μl filtered solutions using ICS-3000 (Dionex, USA).

### Histological analysis

Cecum and spleens from all animals were collected, fixed in 10% neutral buffered formalin for at least 48 h prior to embedding in paraffin and stained with hematoxylin and eosin (H&E). Pathological scores were determined in a blinded manner using a scoring scheme as described previously [34,35].

### Statistical analysis

Statistical analysis was performed using R (version 4.2.1). ANOVA was used to analyze log10-transformed bacterial titers and SCFA concentrations, followed by Tukey’s test. For metabolomics, the normality and homogeneity of variance of metabolites data were checked. Metabolites that met the normal distribution were subjected to ANOVA, and the others were analyzed using Kruskal–Wallis text, followed by *P* value adjustment through FDR. Only metabolites with adjusted *P* < 0.05 were further compared pair-wise with Tukey’s test. Metabolites exhibiting log_2_FC > 0 and VIP score < 1 were identified as biomarkers discriminating different treatments. *P* < 0.05 was considered statistically significant.

## Results

### Dietary fiber deprivation alters the gut microbiome and compromises CR against *S.* Typhimurium infection

**Figure 1:**
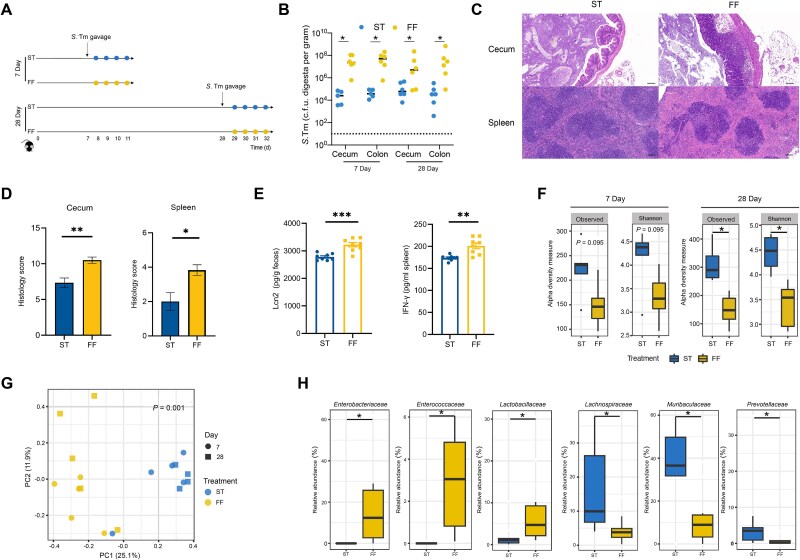
Dietary fiber deprivation compromises colonization resistance against *S.* Typhimurium infection in mice. (A) Schematic depiction of the experiment. Male C57BL/6 J mice of 6–8 weeks were fed either a standard chow (ST) or a FF diet for 7 or 28 days, followed by an infection with *S.* Typhimurium and sample collection at 4 days post-infection (dpi) (*n* = 6–8). (B) The *S.* Typhimurium titers in the cecal and colonic contents of mice at 4 dpi. (C) Representative H&E sections of the cecum and spleen at 4 dpi from mice fed ST or FF food for 28 days. Scale bars represent 100 μm. (D) Composite histopathology score of the cecal and spleen in mice at 4 dpi from mice fed ST or FF food for 28 days. (E) Fecal lipocalin 2 (Lcn2) level and splenic IFN-γ levels at 4 dpi from mice fed ST or FF food for 28 days. (F) Boxplots showing observed species and Shannon index of the colonic microbiota of mice fed either ST or FF diet for 7 and 28 days. (G) Bray–Curtis metrics-based PCoA plot of the colonic microbiota of mice fed either ST or FF diet for 7 and 28 days. (H) Representatives of differentially enriched colonic bacteria in mice fed either ST or FF diet for 28 days. ^*^P < 0.05, ^**^P < 0.01, ^***^P < 0.001.

Dietary fiber is known to influence CR against enteric infections [36]. However, the underlying mechanisms remain largely unexplored. To evaluate the effect of dietary fiber deprivation on the gut microbiome and CR in a murine model of *S.* Typhimurium infection, we fed mice with a standard chow (ST) or a FF diet for 7 or 28 days to represent short-term and long-term effects of dietary fiber deprivation, respectively, followed by an infection with *S.* Typhimurium (Fig. 1A). ST chow comprised soybean meal, corn, and wheat, the FF diet lacked polysaccharides, but included a high level of sucrose. Relative to ST controls, FF mice had a 2–3 log increase in the *S.* Typhimurium titer in both the cecum and colon at 4 dpi, regardless of whether dietary fiber was deprived for 7 or 28 days (Fig. 1B), indicating a pronounced negative impact of fiber deprivation on CR against *S.* Typhimurium. No significant differences were observed in the stool and most organs between the FF and ST groups, mice fed the FF diet for 28 days had significantly increased *S.* Typhimurium in the mesenteric lymph nodes (MLN) compared to the ST mice (Fig. S1A-D). Consistently, H&E analysis revealed more pronounced tissue damage and significantly higher histological scores in the cecum and spleen of FF-fed mice compared to ST controls (Fig. 1C-D). Fecal Lcn2 and splenic IFN-γ levels were significantly elevated in FF-fed mice compared to ST controls (Fig. 1E). This suggests that fiber deprivation exacerbates pathogen burden and systemic inflammation in a murine model of *S.* Typhimurium infection.

To further examine the impact of fiber deprivation on the gut microbiome, we profiled the bacterial community in the colon using 16S rRNA gene amplicon sequencing. No significant difference in baseline microbiome composition was observed in mice prior to dietary intervention (Fig. S2). Both short- and long-term fiber deprivation led to an obvious reduction in bacterial α-diversity (observed species and Shannon index), with the 28-day deprivation showing a more pronounced effect (*P* < 0.05), both before and 4 dpi with *S.* Typhimurium (Fig. 1F, and Fig. S3A-B). Apparently, dietary fiber deprivation caused a significant shift in the bacterial community structure, irrespective of the treatment duration (*P* < 0.001, PERMANOVA, Fig. 1G) with or without *S.* Typhimurium challenge (Fig. S3C). The microbiota composition of FF-fed mice transitioned from being dominated by *Bacteroidetes* and *Firmicutes* to being dominated by *Bacteroidetes*, *Firmicutes*, and *Proteobacteria* (Fig. S4). Specifically, relative abundances of *Enterobacteriaceae*, *Enterococcaceae*, and *Lactobacillaceae* were significantly increased in response to dietary fiber deprivation, the abundances of obligate anaerobic bacteria, such as *Lachnospiraceae*, *Muribaculaceae*, and *Prevotellaceae,* were significantly decreased (Fig. 1H). Consistently, the relative abundance of *Muribaculaceae* significantly decreased in FF-fed mice without *S.* Typhimurium challenge (Fig. S3D).

### Reversion to standard diet fully restores CR against *S.* Typhimurium infection

**Figure 2:**
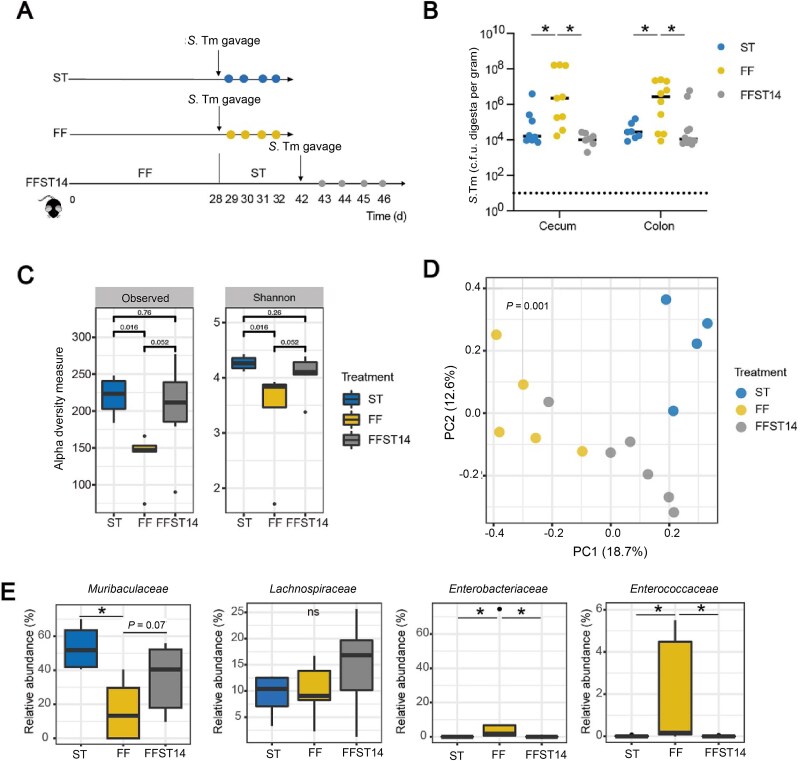
Reversion to standard diet restores colonization resistance against *S.* Typhimurium infection. (A) Schematic depiction of the experiment. Mice were fed either a ST or a FF diet for 28 days, followed by an infection with *S.* Typhimurium and sample collection at 4 dpi (*n* = 10). A third group of mice (FFST14) were fed the FF diet for 28 days and switched back to the ST diet for another 14 days prior to *S.* Typhimurium infection. (B) The *S.* Typhimurium titers in the cecal and colonic contents among three groups of mice at 4 dpi. (C) Boxplots showing observed species and Shannon index of the colonic microbiota among three groups of mice at 4 dpi. (D) Bray–Curtis metrics-based PCoA plot of the colonic microbiota among three groups of mice at 4 dpi. (E) Representatives of differentially enriched colonic bacteria among three groups of mice at 4 dpi. ^*^P < 0.05; n.s., not significant.

To further explore the causal relationship between dietary fiber deprivation and CR against *S.* Typhimurium, we fed mice with FF-diet for 28 days and then switched to the ST diet for another 14 days (Fig. 2A). To our excitement, mice fully regained CR compared to the ST-fed mice (Fig. 2B). No significant differences in the *S.* Typhimurium titer were observed in the stool and organs among different groups of mice (Fig. S5).

Reversion to the ST diet largely restored α-diversity (*P* = 0.26, Fig. 2C), but not β-diversity (*P* = 0.001, PERMANOVA, Fig. 2D), of the intestinal bacterial community. The mouse colon was dominated by *Muribaculaceae, Lachnospiraceae*, and *Ruminococcaceae* (Fig. S6). Consistently, *Enterobacteriaceae*, *Enterococcaceae* bloomed in response to dietary fiber deprivation. Although depleted significantly in FF mice, *Muribaculaceae* was obviously restored upon switching back to the ST diet (Fig. 2E). *Lachnospiraceae* and *Prevotellaceae* remained unchanged. This suggested that *Muribaculaceae* may be a key bacterial family in mediating CR against *S.* Typhimurium.

### Fecal microbiota transplantation identifies *Muribaculum intestinale* as a candidate bacterium mediating CR against *S.* Typhimurium

**Figure 3:**
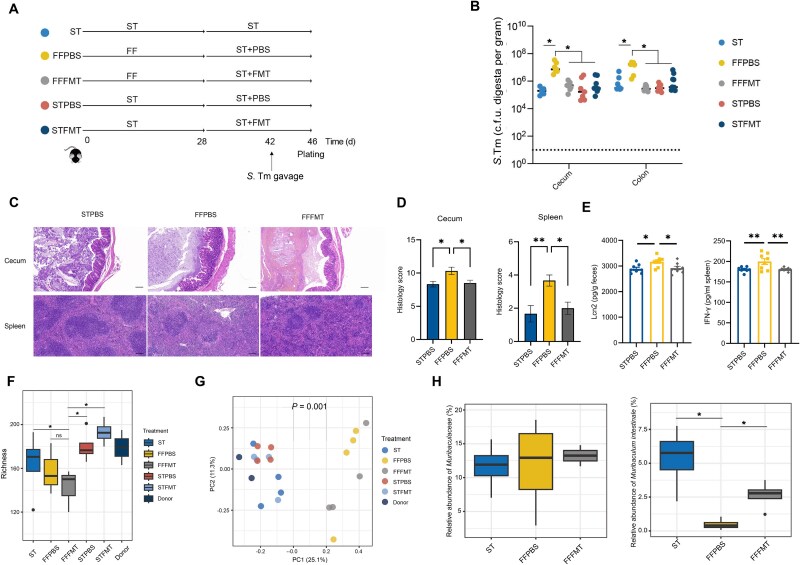
Fecal microbiota transplantation identifies *M. intestinale* as key bacterium mediating colonization against *S.* Typhimurium. (A) Schematic scheme of the experiment. Mice were fed either a standard chow (ST) or a FF diet (*n* = 6–8). On Day 28, they were gavaged daily with PBS or fecal microbiota of ST-fed mice (FMT) daily for 14 days, followed by an infection with *S.* Typhimurium on Day 42 and sample collection at 4 days post-infection (dpi). (B) The *S.* Typhimurium titers in the cecal and colonic contents of mice at 4 dpi. (C) Representative H&E sections of the cecum and spleen at 4 dpi. Scale bars represent 100 μm. (D) Composite histopathology scores of the cecum and spleen 4 dpi. (E) Fecal lipocalin 2 (Lcn2) and splenic IFN-γ levels at 4 dpi. (F) Boxplots showing observed species of the colonic microbiota of mice at 4 dpi. (G) Bray–Curtis metrics-based PCoA plot of the colonic microbiota of mice at 4 dpi. (H) Relative abundances (%) of *Muribaculaceae* and *M. intestinale* in the colon of mice at 4 dpi. ^*^P < 0.05, ^**^P < 0.01; n.s., not significant.

To identify specific bacteria involved in CR against *S.* Typhimurium, we transplanted the fecal microbiota of ST-fed mice to mice fed the ST or FF diet for 28 days (Fig. 3A). FMT conferred *S.* Typhimurium resistance to FF-fed mice in both the cecum and colon (Fig. 3B). Histological analysis revealed that FMT reduced tissue damage in the cecum and spleen, with significantly lower histological scores (Fig. 3C-D). Additionally, the levels of Lcn2 in the feces and IFN-γ in the spleen significantly decreased in FF-fed mice receiving FMT (Fig. 3E), further indicating the protective effect of FMT against *S.* Typhimurium infection. Bacterial richness remained reduced in FF mice after FMT (Fig. 3F). FMT failed to restore the bacterial community structure of FF-fed mice to normal (Fig. 3G), supporting the notion that dietary fiber-mediated CR against *S.* Typhimurium may not rely on the restoration of the entire microbial community, and specific commensal bacteria may be involved. As expected, the colonic microbiome in mice was highly similar among different groups of mice before dietary intervention or FMT (Fig. S7), FMT contributed to restore the bacterial diversity and community structure in FF-fed mice without *S.* Typhimurium challenge (Fig. S8A-C). Differential enrichment analysis further revealed that the *Muribaculaceae* family remained unchanged after FMT gavage (Fig. 3H). Additionally, *Muribaculum intestinale,* a member of *Muribaculaceae*, was depleted in response to dietary fiber deprivation and partially recovered following FMT, irrespective of *S.* Typhimurium challenge (Fig. 3H and Fig. S8D), suggesting that *M. intestinale* may be a candidate bacterium involved in CR to *S.* Typhimurium.

### Succinate and propionate are differentially enriched in dietary fiber-deprived mice

To assess the potential of *M. intestinale* in fighting against *S.* Typhimurium infection, we retrieved the *M. intestinale* genome from our metagenomic sequences and annotated it through alignment with eggNOG database. We found that the *M. intestinale* genome encodes genes involved in the biosynthesis of secondary metabolites, biosynthesis of amino acids, pyrimidine metabolism, oxidative phosphorylation, and microbial metabolism in diverse environments (Fig. S9). Utilizing gutSMASH, we further identified genes within the *M. intestinale* genome participating in metabolic pathways responsible for fumarate-succinate and succinate-propionate conversions (Fig. S10). This suggests *M. intestinale* may confer resistance to *S.* Typhimurium infection through the production or conversion of specific metabolites.

**Figure 4:**
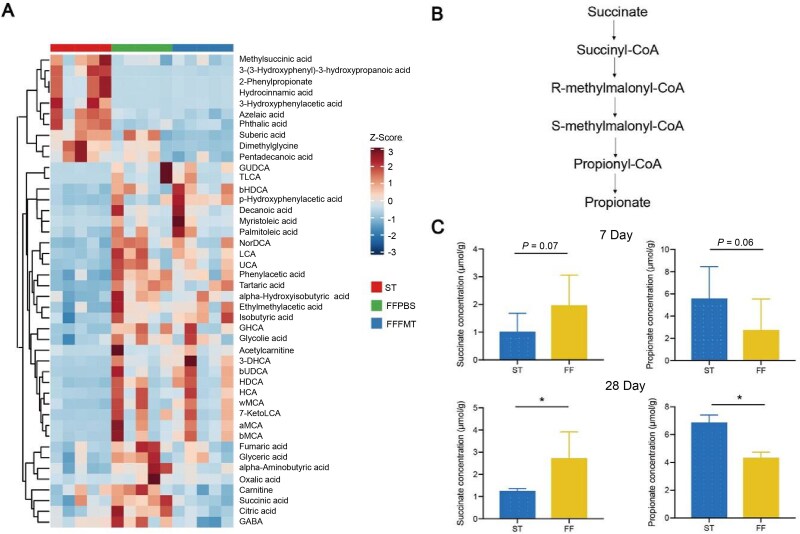
Targeted metabolomics identifies succinate and propionate as potential metabolites mediating colonization resistance against *S.* Typhimurium. (A) Differentially enriched metabolites identified by targeted metabolomics. (B) The metabolic pathway of converting succinate into propionate. (C) Influence of 7- or 28-day dietary fiber deprivation on succinate and propionate concentrations in the feces of mice (*n* = 5). ^*^P < 0.05.

To validate our genomic predictions, targeted metabolomics was performed on samples from the FMT trial, and we identified clusters of organic acids and bile acids to be increased in the FF mice, but only organic acids were restored to normal levels following transplantation with fecal microbiota from ST-fed mice (Fig. 4A). These differentially enriched organic acids are involved in glyoxylate and dicarboxylate metabolism, the tricarboxylic acid (TCA) cycle, alanine, aspartate, and glutamate metabolism (Fig. S11). *S.* Typhimurium is known to perform a complete oxidative TCA cycle and utilize succinate to support its growth in the intestinal tract [37]. Consistently, the intermediates participating in the TCA cycle, such as fumarate, succinate, citrate, and GABA, increased in response to dietary fiber deprivation. It is, therefore, reasonable to speculate that fiber deficiency-induced depletion of *M. intestinale* leads to an accumulation of succinate and activation of the TCA cycle, providing growth advantages for *S.* Typhimurium.

Succinate is well-known to be predominantly converted into propionate as the end product in the intestinal tract [38] (Fig. 4B). To examine the alterations of succinate and propionate in response to dietary fiber deprivation, we measured the concentrations of both acids in fecal samples of mice fed either ST or FF diet for 7 or 28 days. Succinate was significantly increased, although propionate was significantly reduced in response to 28-day dietary fiber deprivation, with 7-day fiber deprivation showing a similar, but a less significant trend (Fig. 4C). Collectively, we revealed that an imbalance between succinate and propionate synthesis may be responsible for compromised CR to *S.* Typhimurium in the context of dietary fiber deficiency.

### *M. intestinale* maintains CR against *S.* Typhimurium by converting succinate into propionate

**Figure 5:**
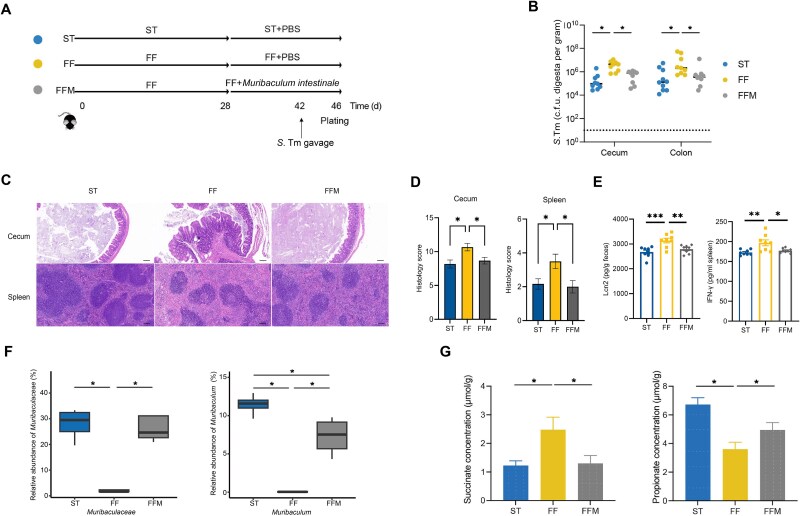
*M. intestinale* confers colonization resistance against *S.* Typhimurium by converting succinate to propionate. (A) Schematic scheme of the experiment. Mice were fed either a ST or a FF diet (*n* = 10). On Day 28, they were gavaged with PBS or 1 × 10 [8] CFU *M. intestinale* daily for 14 days, followed by an infection with *S.* Typhimurium on Day 42 and sample collection at 4 dpi. (B) The *S.* Typhimurium titers in the cecal and colonic contents of mice at 4 dpi. (C) Representative H&E sections of the cecum and spleen. Scale bars represent 100 μm. (D) Composite histopathology scores of the cecum and spleen at 4 dpi. (E) Fecal Lcn2 and splenic IFN-γ levels. (F) Relative abundances (%) of *Muribaculaceae* and *Muribaculum* in the colon among three groups of mice at 4 dpi. (G) Impact of *M. intestinale* administration on succinate and propionate concentrations in the colon of mice at 4 dpi. ^*^P < 0.05, ^**^P < 0.0.

To directly evaluate whether *M. intestinale* is capable of protecting against *S.* Typhimurium infection through modulating production of succinate and propionate, we inoculated FF-fed mice with *M. intestinale* for 14 days, followed by *S.* Typhimurium infection (Fig. 5A). We found that *M. intestinale* administration completely restored CR against *S.* Typhimurium in both the cecum and colon, as evidenced by decreased *S.* Typhimurium titer, minimal histological damage in the cecum and spleen, and reduced levels of Lcn2 and IFN-γ (Fig. 5B-E). Again, no significant difference in the baseline microbiome was observed among different groups of mice before *M. intestinale* administration (Fig. S12). As expected, bacterial richness remained increased in FF mice after oral gavage of *M. intestinale* in the absence of *S.* Typhimurium challenge (Fig. S13A-C). Specifically, both *Muribaculaceae* and *Muribaculum* were enriched in response to *M. intestinale* administration, with or without *S.* Typhimurium challenge (Fig. 5F and Fig. S13D). To further examine whether *M. intestinale* administration could restore the metabolism of succinate and propionate, we measured the fecal concentrations of succinate and propionate in FF-fed mice following daily gavage of *M. intestinale* for 14 days. Both succinate and propionate were restored to normal (Fig. 5G). These results strongly suggested that *M. intestinale* may confer resistance to *S.* Typhimurium by converting succinate to propionate. However, given the functional redundancy of the gut microbiota, it is also possible that other species may cooperate with *M. intestinale* in regulating these metabolic pathways.

**Figure 6:**
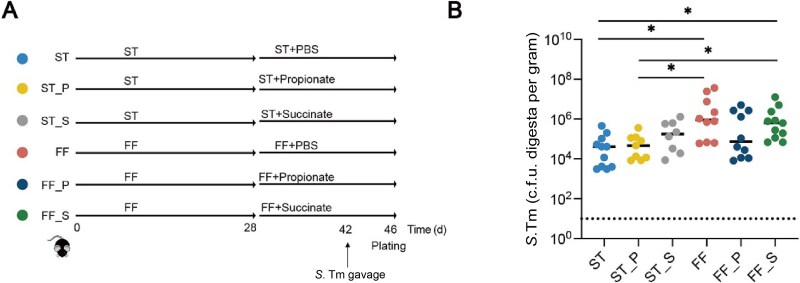
Efficacy of succinate and propionate against *S.* Typhimurium infection. (A) Schematic scheme of the experiment. Mice were fed either a ST or a FF diet for 42 days (*n* = 10). On Day 28, they were gavaged with PBS, 10 mg/day propionate, or 10 mg/day succinate daily for 14 days, followed by an infection with *S.* Typhimurium on Day 42 and sample collection at 4 dpi. (B) The *S.* Typhimurium titers in the cecal and colonic contents of mice at 4 dpi. ^*^P < 0.05.

To further confirm the role of succinate and propionate in protection against *S.* Typhimurium, we administered succinate or propionate by oral gavage for 14 days after mice were fed either ST or FF diet (Fig. 6A). Propionate partially suppressed *S.* Typhimurium colonization in FF-fed mice, although succinate failed (Fig. 6B), suggesting that *M. intestinale* may modulate *S.* Typhimurium colonization by converting succinate to propionate when dietary fiber is deprived.

## Discussion

Dietary fiber serves as a primary energy source for intestinal microbiota. Inadequate dietary fiber intake can result in an insufficient nutrient supply for intestinal microbiota, causing dysbiosis [14,39]. Intestinal dysbiosis has been linked to pathogen susceptibility, inflammatory bowel disease, and cognitive impairments [40–43]. However, the mechanisms by which dietary fiber deficiency affects CR remains unclear. In this study, we utilized a mouse *Salmonella* infection model to explore the impact of dietary fiber deprivation on CR. Through dietary reversion, FMT, metabolomics, and targeted administration of *M. intestinal*, succinate, and propionate, we uncovered that *M. intestinale* confers resistance to *S.* Typhimurium infection in fiber-deprived mice by converting succinate into propionate.

Dietary fiber deprivation was shown to abolish fiber-degrading bacteria in a humanized mouse model colonized with 14 bacterial strains, resulting in enhanced intestinal inflammation and reduced CR to *C. rodentium* [15]. Another study with conventional mice showed diminishment of fiber-degrading bacteria *Muribaculaceae* and *Prevotellaceae*, but enrichment of *Streptococcaceae, Clostridiales Family XIII*, *Desulfovibrionaceae*, and *Burkholderiaceae* in response to fiber deprivation [37]. Consistently, we observed that dietary fiber deprivation led to a dramatic reduction in the abundance of *Muribaculaceae* and *Lachnospiraceae*, two most dominant bacteria in the mouse intestinal tract, suggesting the potential importance of fiber-degrading bacteria in maintaining CR.

Dietary fiber-degrading bacteria primarily utilize various carbohydrate-active enzyme systems to degrade fiber and produce small-molecule metabolites, mainly SCFAs [44]. Previous studies annotating 153 *Muribaculaceae* genomes found a substantial representation of carbohydrate-active enzymes, particularly glycoside hydrolase families GH13 and GH43, indicating their involvement in starch, arabinoxylan, and xylan degradation [45]. Our study highlighted that *Muribaculaceae*, with its significant carbohydrate-active enzyme encoding potential, may play a pivotal role in dietary fiber degradation in the mouse gut [46]. The lack of dietary fiber disrupts the supply of carbohydrates, leading to the extinction of *Muribaculaceae* in the gut and underscoring the association between *Muribaculaceae* depletion and *S.* Typhimurium CR.

SCFAs, particularly propionate, are crucial metabolites in maintaining CR [47]. Propionate has been shown to influence resistance to *Salmonella* by disrupting the pH balance within *Salmonella* cells [48]. Our findings revealed that a lack of dietary fiber leads to decreased synthesis of propionate associated with an increase in succinate. As the main precursor for propionate synthesis, intestinal succinate is continuously converted to propionate under normal physiological conditions [38]. However, dysbiosis, as observed in dietary fiber deficiency, disrupts the equilibrium between succinate production and consumption among commensal bacteria. A recent study demonstrated that *M. intestinale* YL27 induces antigen-specific cytokine responses through a metabolite known as MiCL-1 [49]. Our annotation of the metabolic potential of the *M. intestinale* genome indicated its capability to convert succinate to propionate. Thus, the loss of *M. intestinale* induced by dietary fiber deficiency reduces the ability of the host to convert succinate into propionate, resulting in succinate accumulation. Increased succinate levels has been linked to enhanced infection by *Clostridium difficile* and *C. rodentium*, highlighting the potential role of succinate in promoting pathogen colonization [50,51]. The observed dysbiosis and associated loss of propionate production highlight the importance of these bacteria in maintaining CR and regulating gut metabolism.

Based on the results of previous studies, an intact TCA cycle is essential for *Salmonella* infection in mice, and the absence of succinate dehydrogenase/fumarate reductase in the TCA cycle can reduce *Salmonella* colonization [37,52]. Therefore, we propose that *M. intestinale*–mediated conversion of succinate to propionate disrupts the completion of a TCA cycle, leading to a survival disadvantage to *Salmonella*. Whereas our findings indicate that *M. intestinale* restores propionate levels and confers protection against *S.* Typhimurium infection, we cannot exclude the possibility that other members of the microbiota, particularly low-abundance bacteria, may also contribute to this process. Further studies, such as challenging germ-free mice with *M. intestinale* and *Salmonella*, are necessary to determine whether *M. intestinale* acts independently or in cooperation with other commensal bacteria to confer CR.

Our findings suggest that *M. intestinale* plays a significant role in restoring CR by converting succinate into propionate. Specifically, propionate was able to maintain CR against *S.* Typhimurium, whereas succinate showed no apparent beneficial effect. However, a definitive conclusion on the role of succinate-propionate conversion in CR against *Salmonella* requires the generation of an *M. intestinale* strain deficient in a key enzyme in this pathway. Further studies are warranted to confirm the significance of the succinate-propionate pathway in CR against *Salmonella* infection.

## Conclusion

This study aimed to investigate the microbiota-mediated CR against *Salmonella*. We demonstrated that the depletion of *M. intestinale* induced by dietary fiber deficiency contributes to a reduction in CR against *Salmonella*. Additionally, we demonstrated that the administration of *M. intestinale* restores CR in mice subjected to a FF diet. Dietary fiber deficiency leads to an accumulation of succinate and a concurrent reduction in propionate synthesis. The capacity of *M. intestinale* to convert succinate to propionate emerges as a potential mechanism underlying its resistance to *Salmonella* colonization in the context of dietary fiber deficiency. Further studies are needed to investigate the potential involvement of other commensal bacteria and the necessity of the succinate-to-propionate conversion pathway in CR against *Salmonella* infection.

## Supplementary Material

Figure_S1_wraf069

Figure_S2_wraf069

Figure_S3_wraf069

Figure_S4_wraf069

Figure_S5_wraf069

Figure_S6_wraf069

Figure_S7_wraf069

Figure_S8_wraf069

Figure_S9_wraf069

Figure_S10_wraf069

Figure_S11_wraf069

Figure_S12_wraf069

Figure_S13_wraf069

Supplementary_Table_S1_wraf069

supplementary_information_4_8_wraf069

## Data Availability

All sequencing reads have been deposited at the NCBI sequence read archive under BioProject #PRJNA1225023 at https://www.ncbi.nlm.nih.gov/bioproject/?term=PRJNA1225023.
