# Supplementary figures and images for: Muribaculum intestinale restricts Salmonella Typhimurium colonization by converting succinate to propionate
Source: ISME J. 2025 Apr 18;19(1):wraf069. doi: 10.1093/ismejo/wraf069 (PMC12064562; doi:10.1093/ismejo/wraf069)

**A**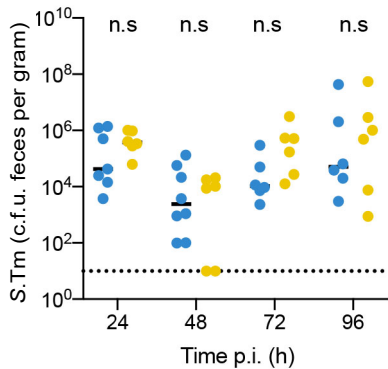

● ST ● FF

**B**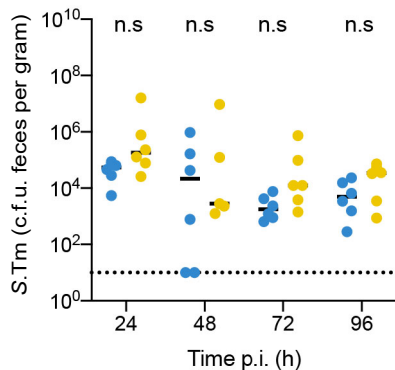**C**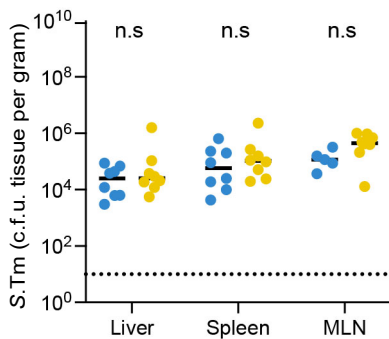**D**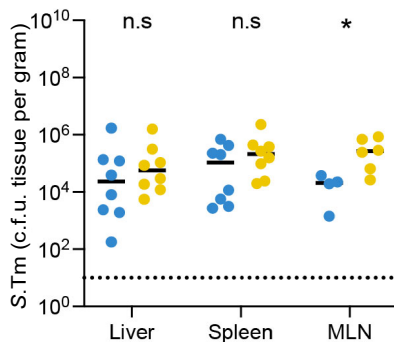

Supplement: Figure_S1_wraf069 [file figure_s1_wraf069.pdf]

**A**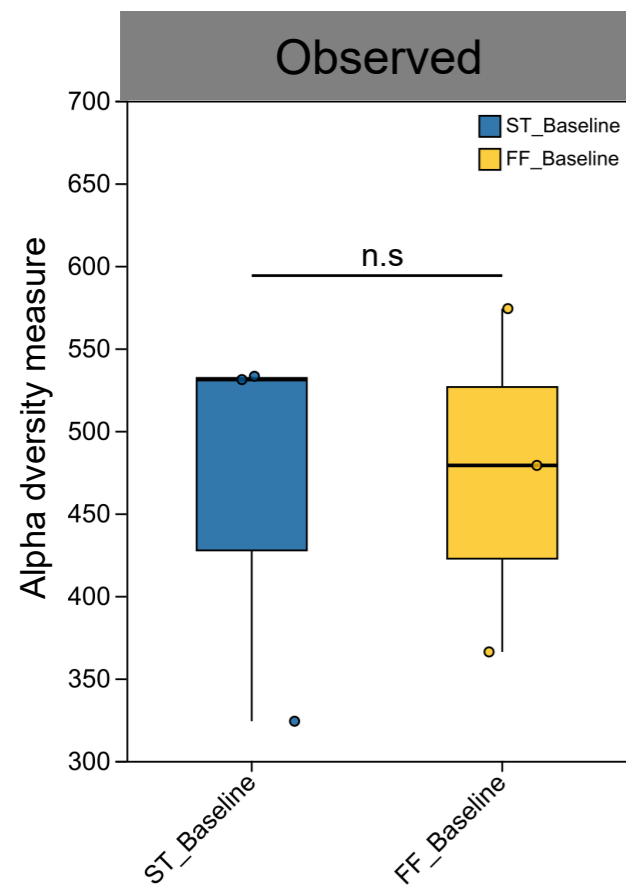**B**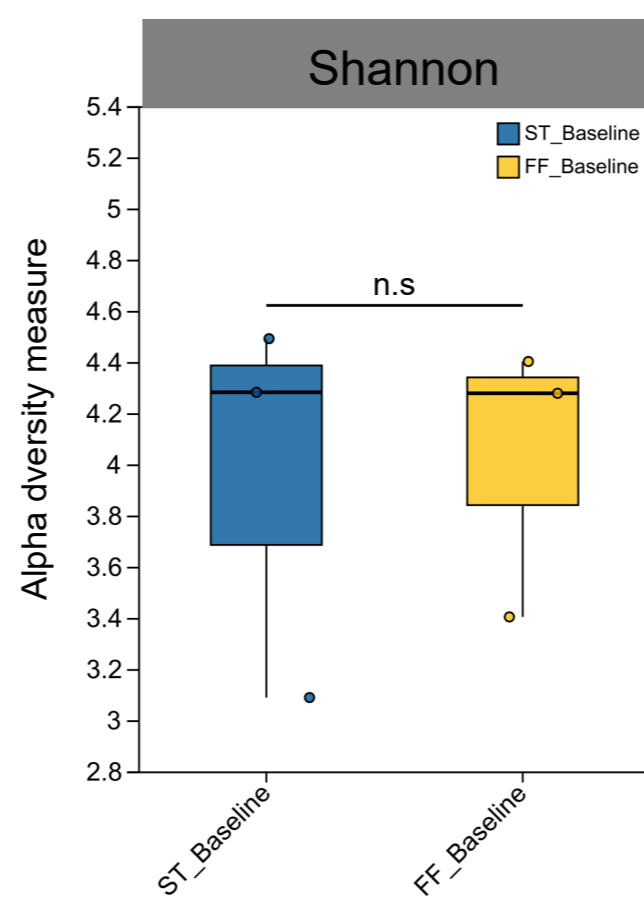**C**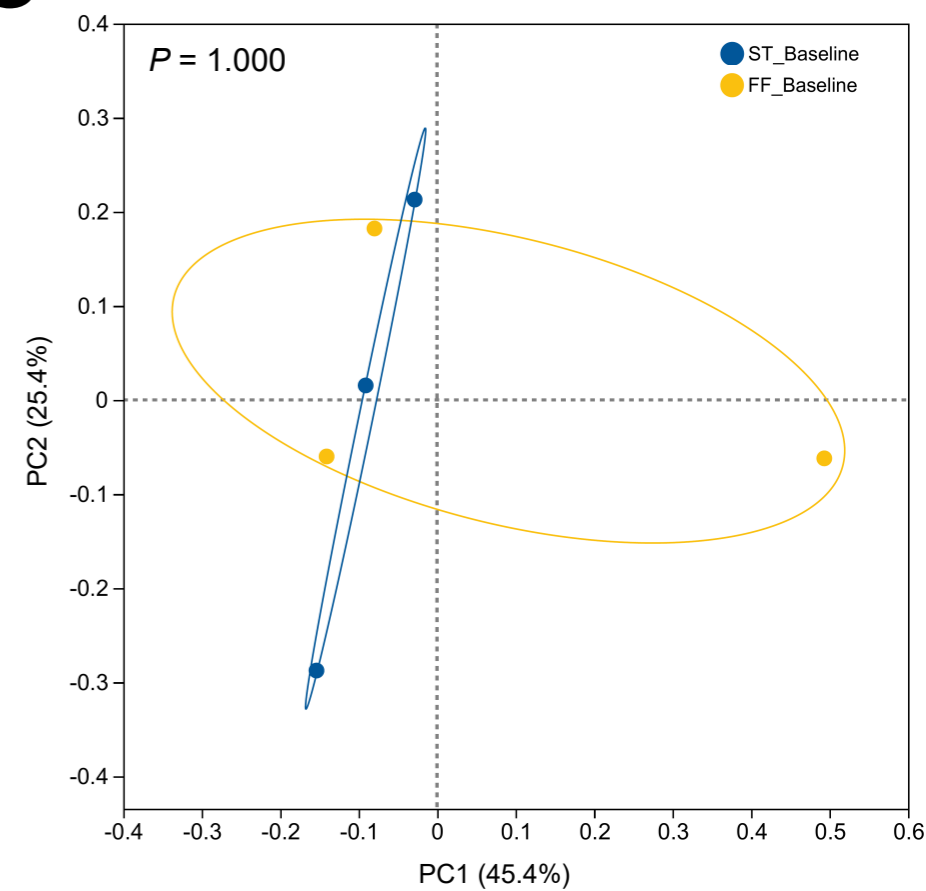**D**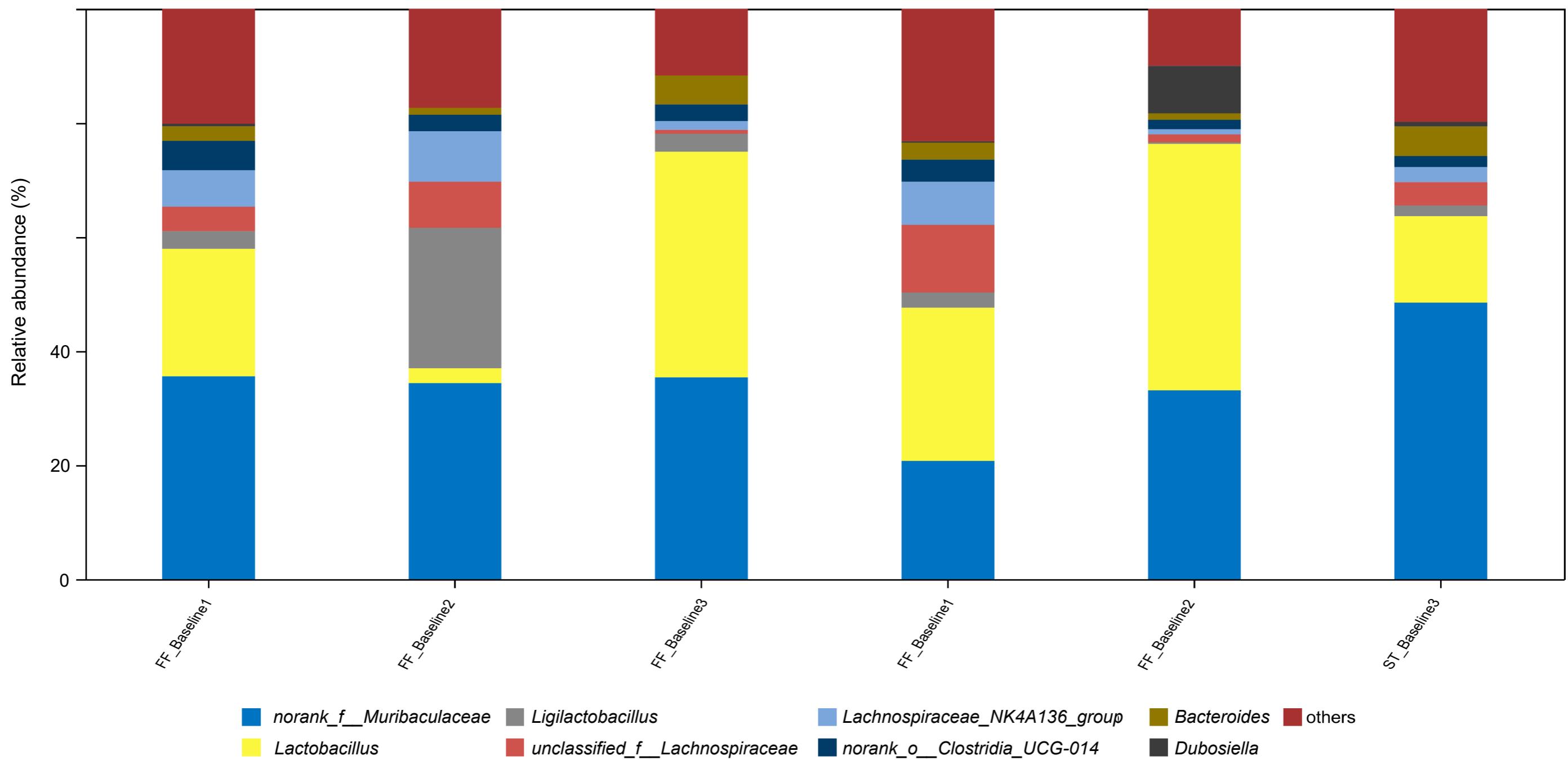

Supplement: Figure_S2_wraf069 [file figure_s2_wraf069.pdf]

**A**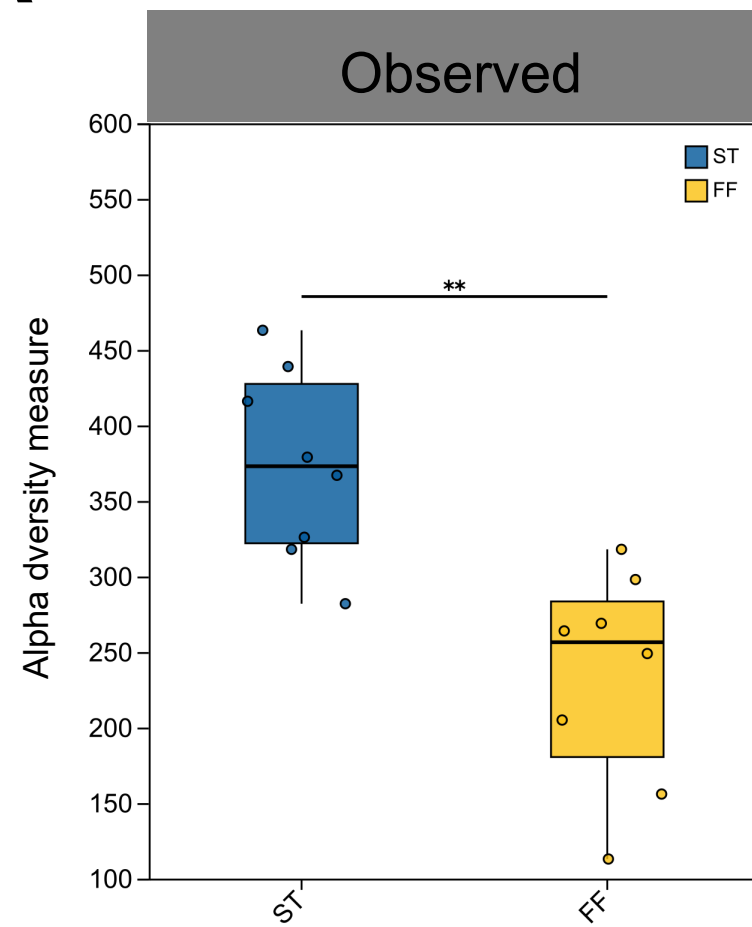**B**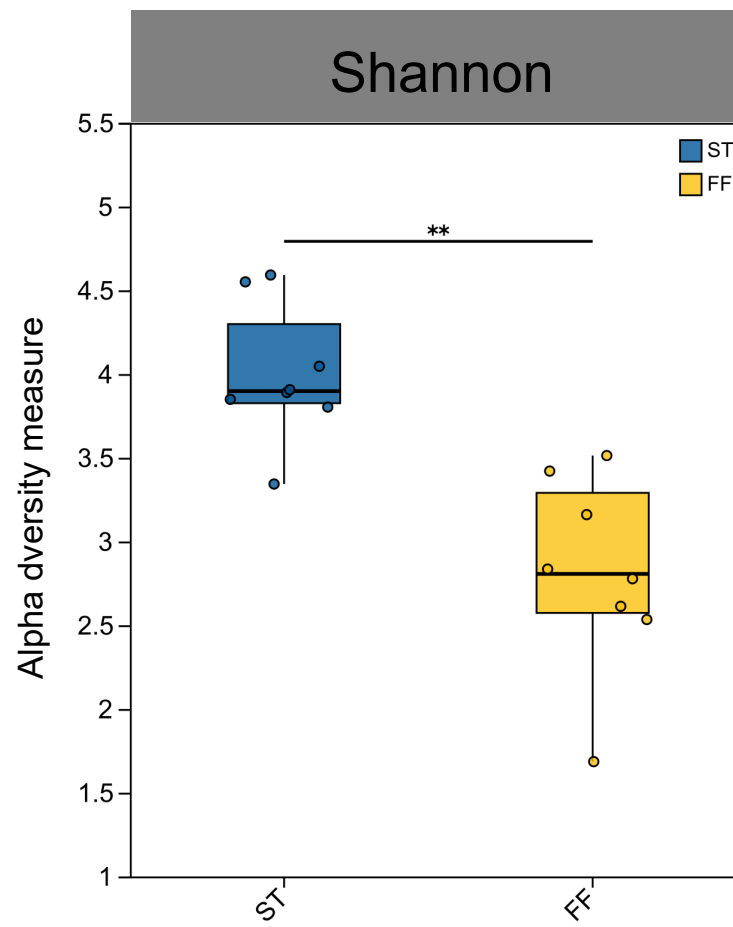**C**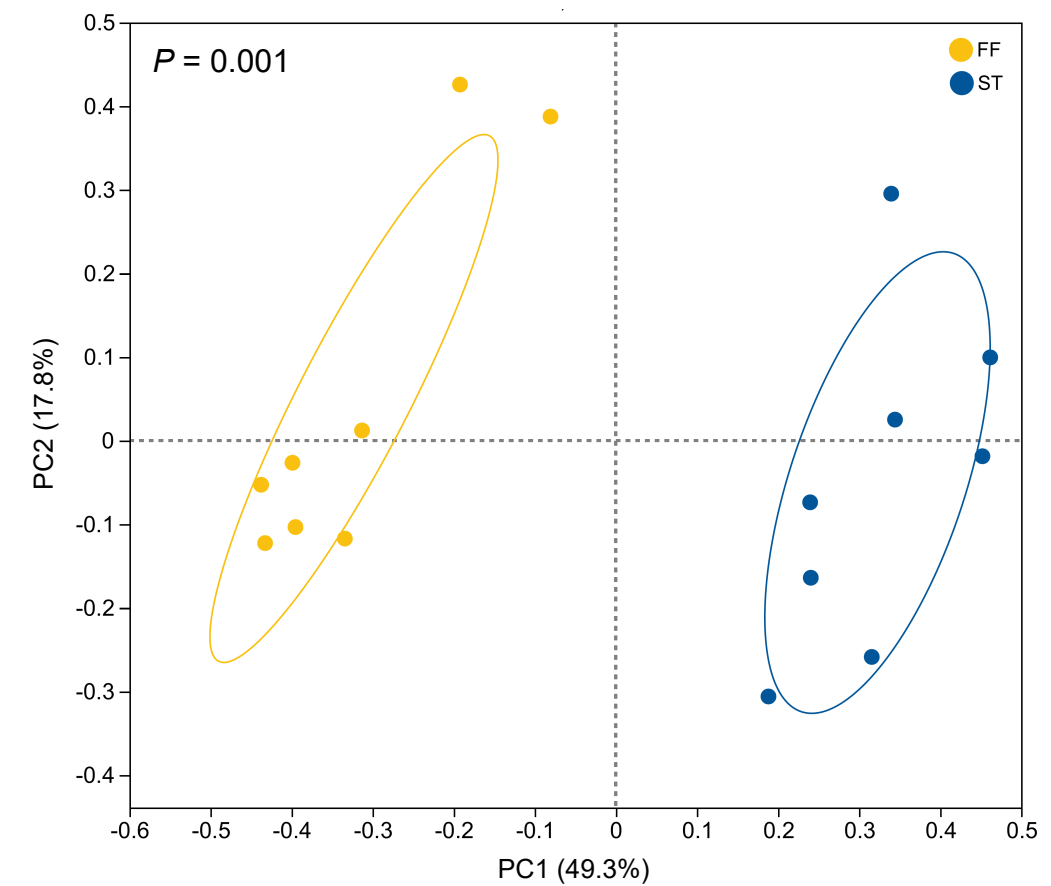**D**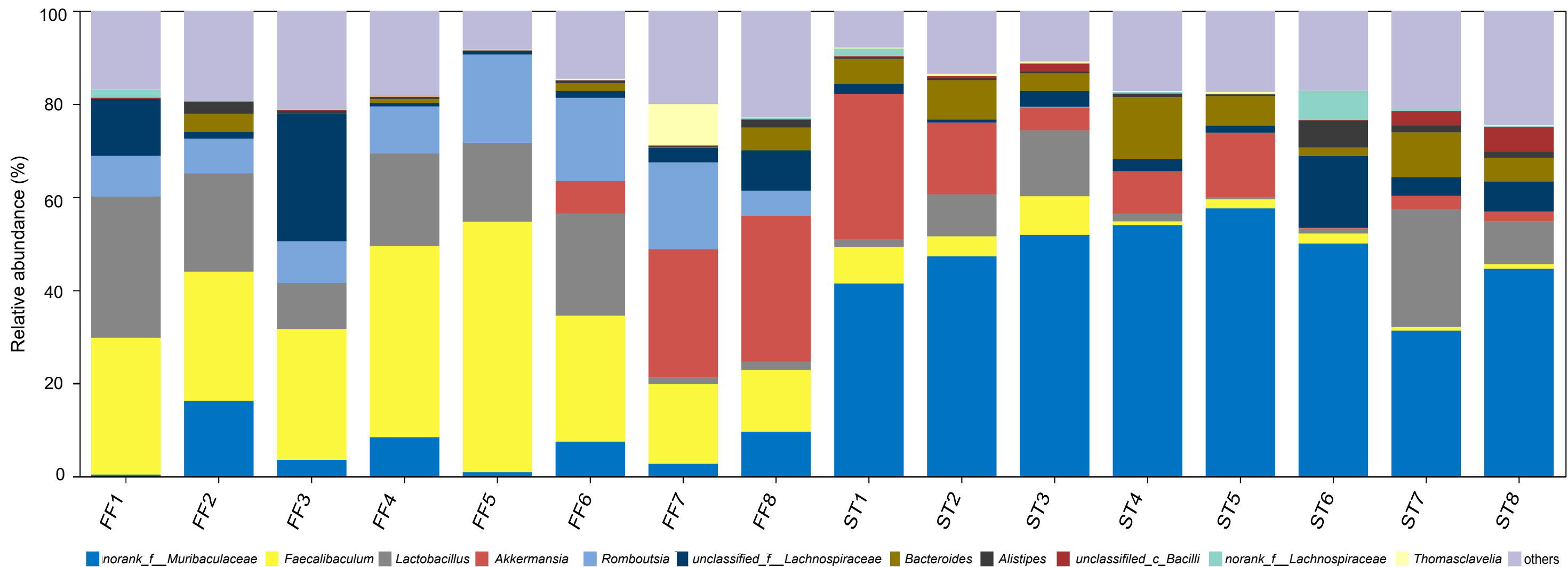

Supplement: Figure_S3_wraf069 [file figure_s3_wraf069.pdf]

A

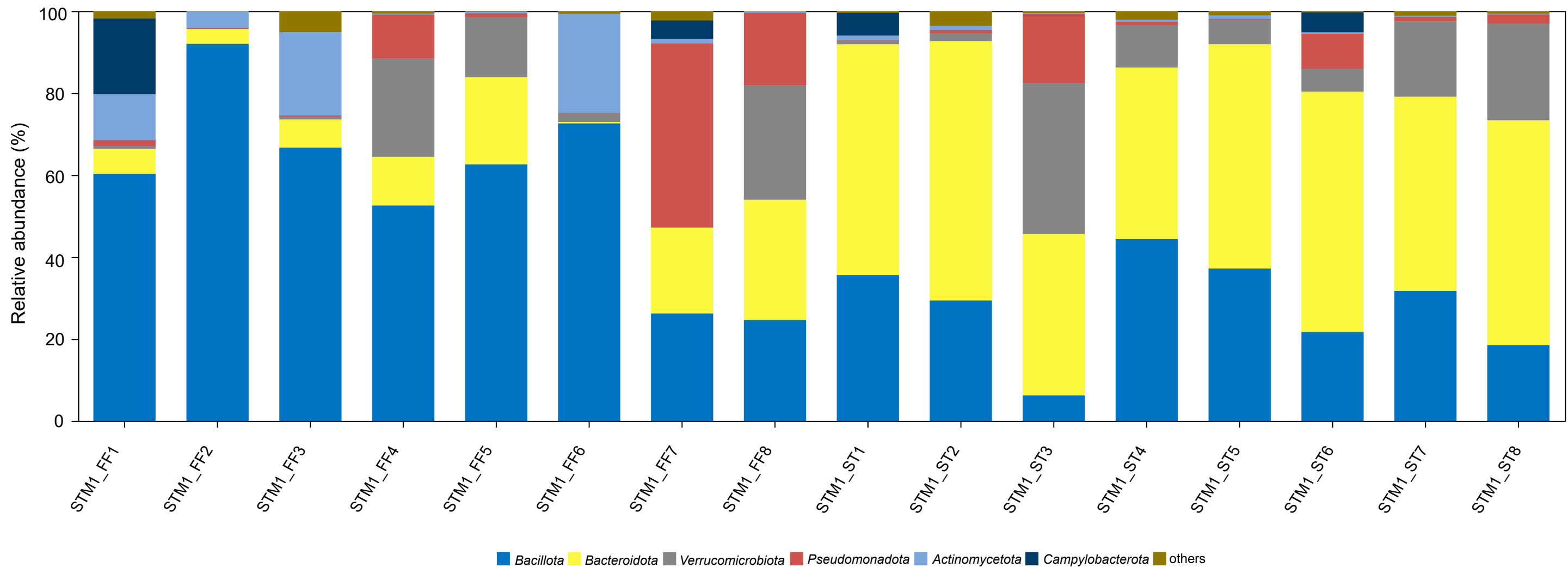

B

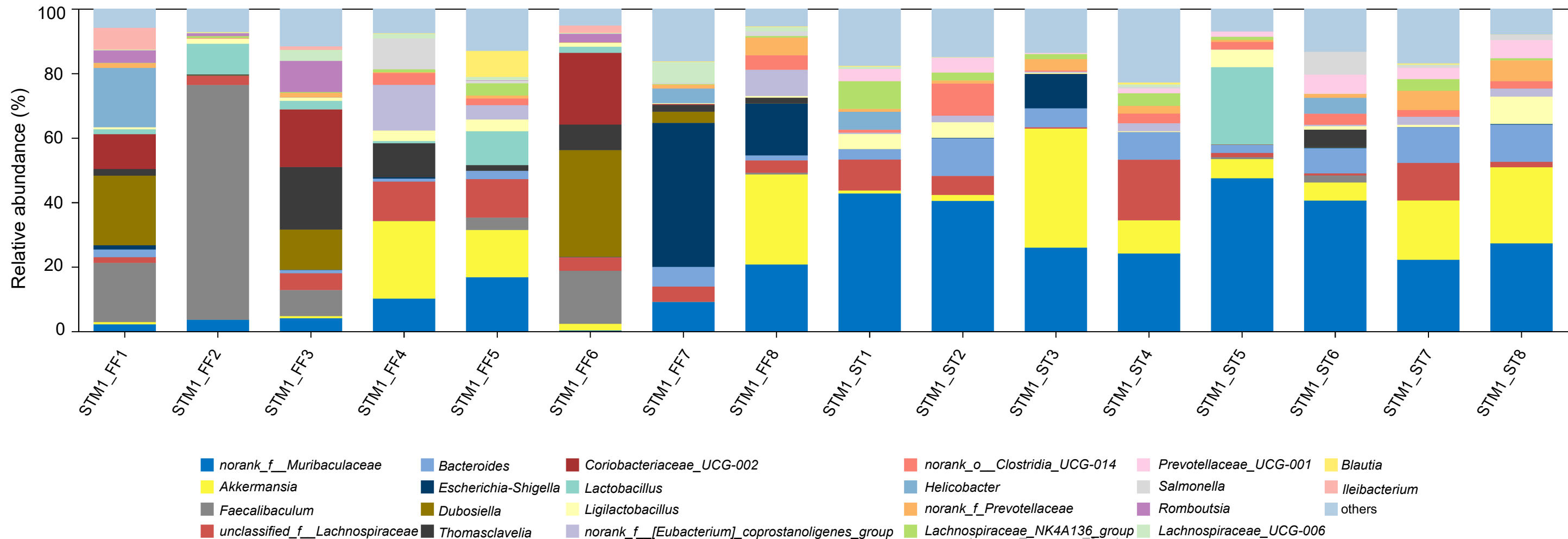

Supplement: Figure_S4_wraf069 [file figure_s4_wraf069.pdf]

**A**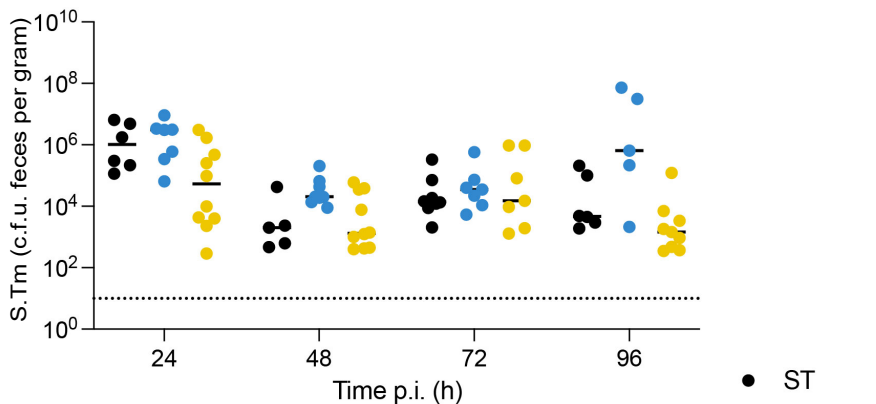**B**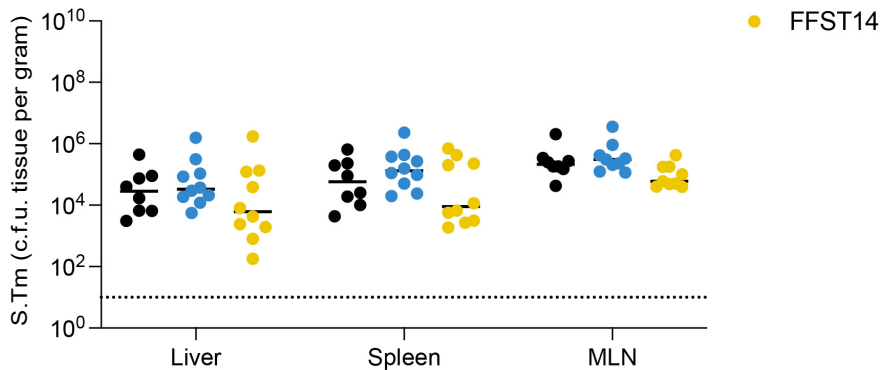

Supplement: Figure_S5_wraf069 [file figure_s5_wraf069.pdf]

A

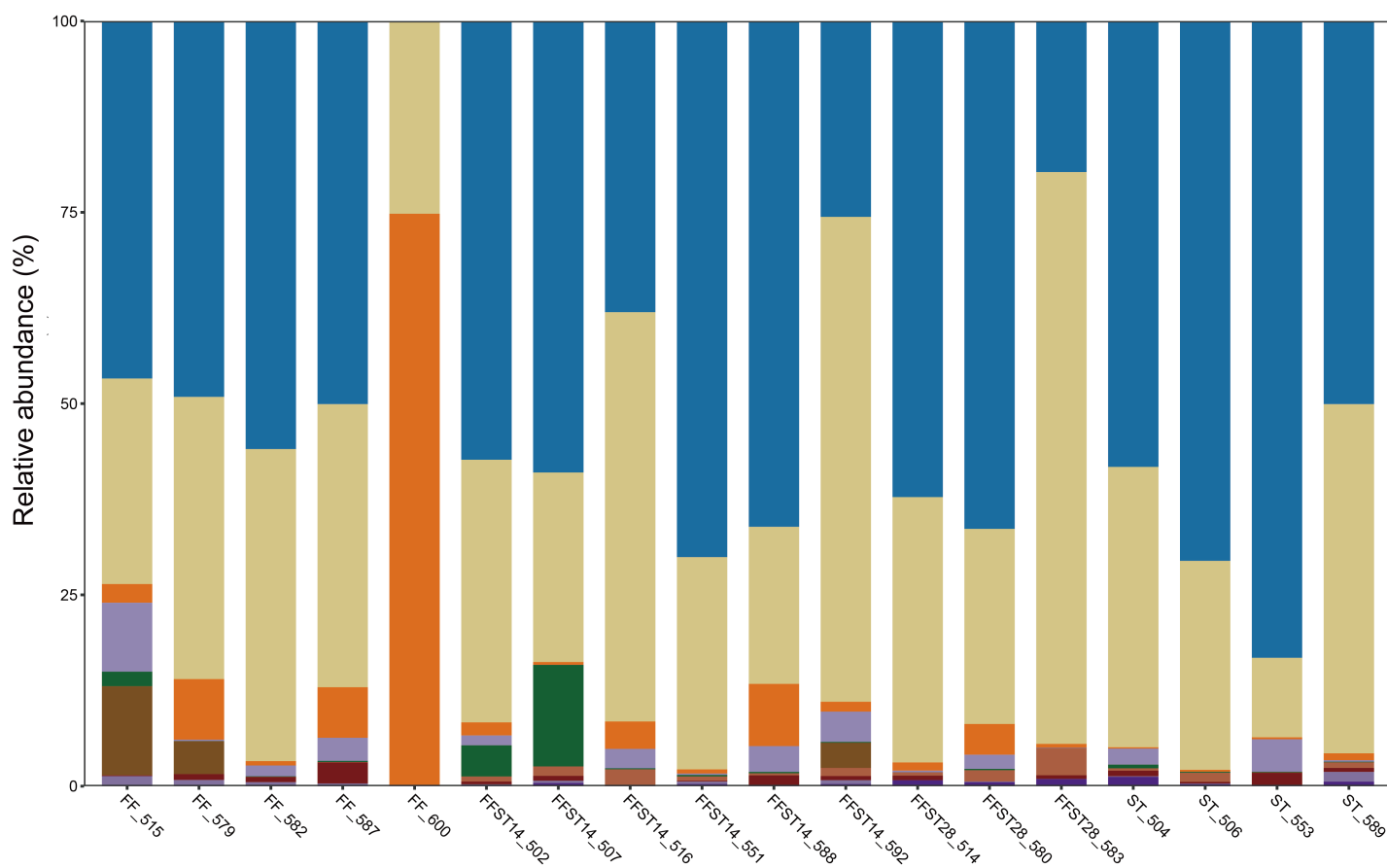

B

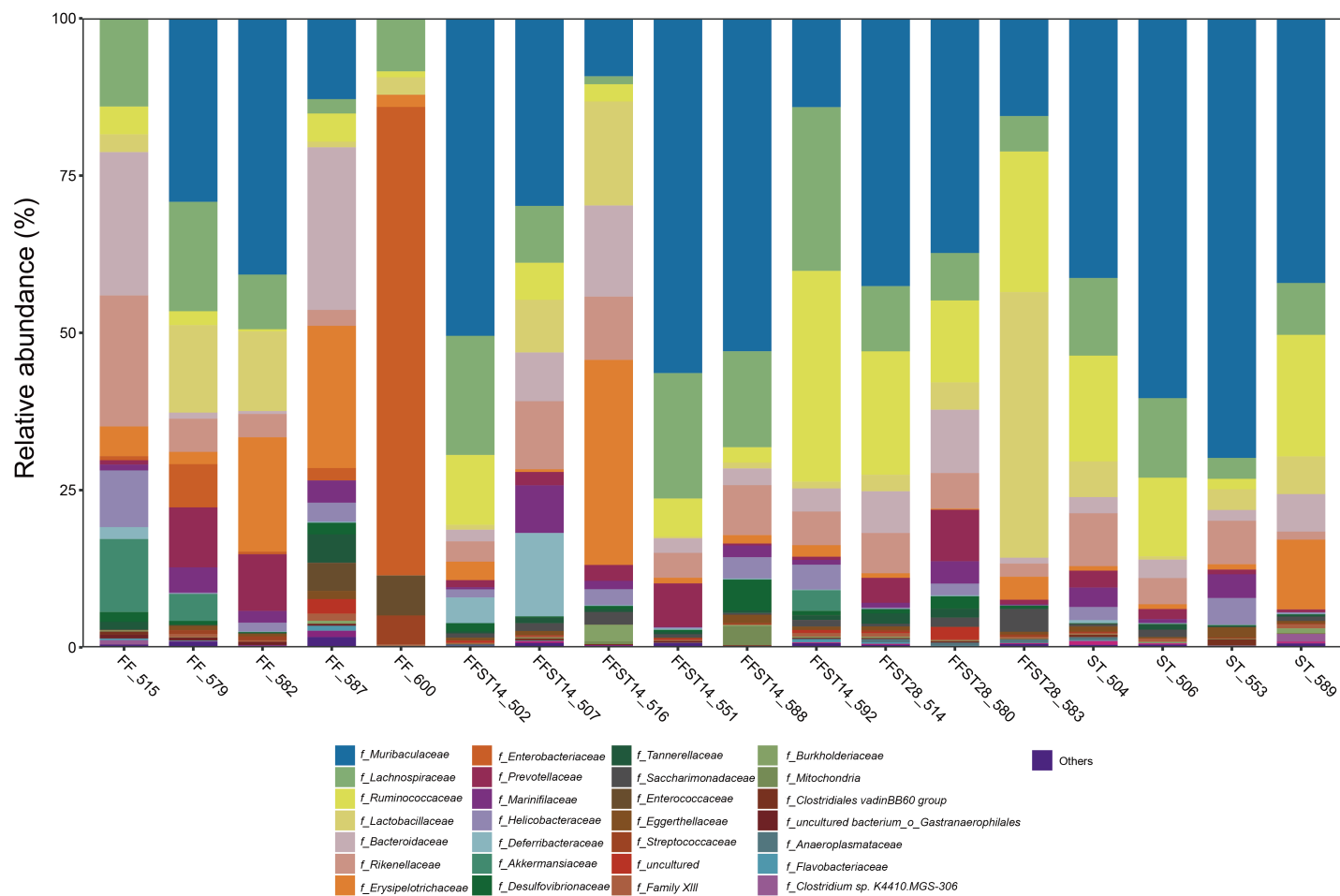

Supplement: Figure_S6_wraf069 [file figure_s6_wraf069.pdf]

**A**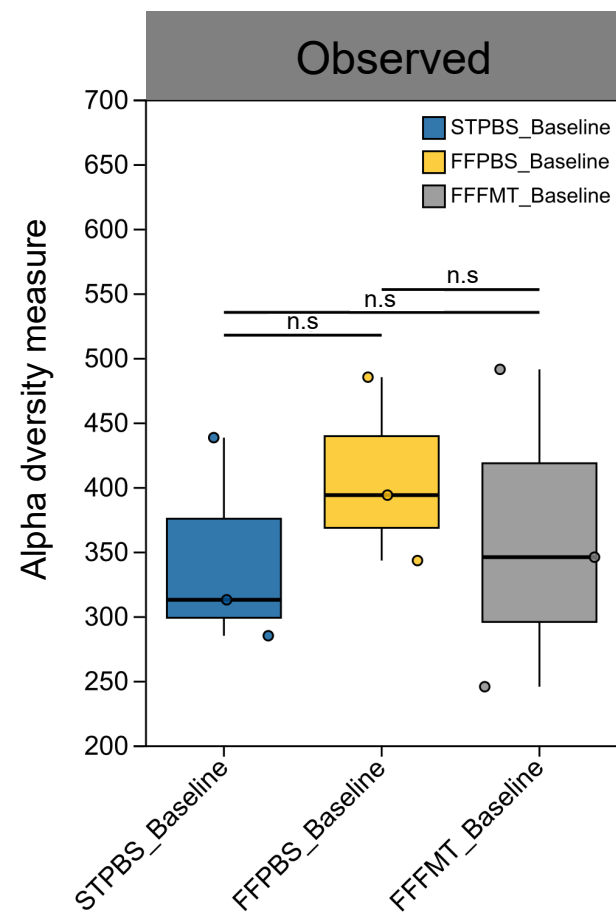**B**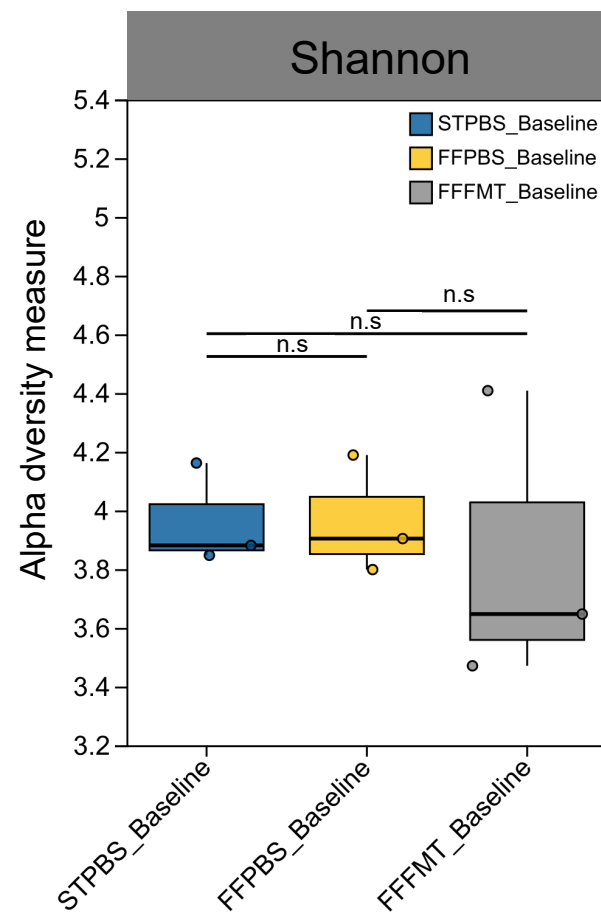**C**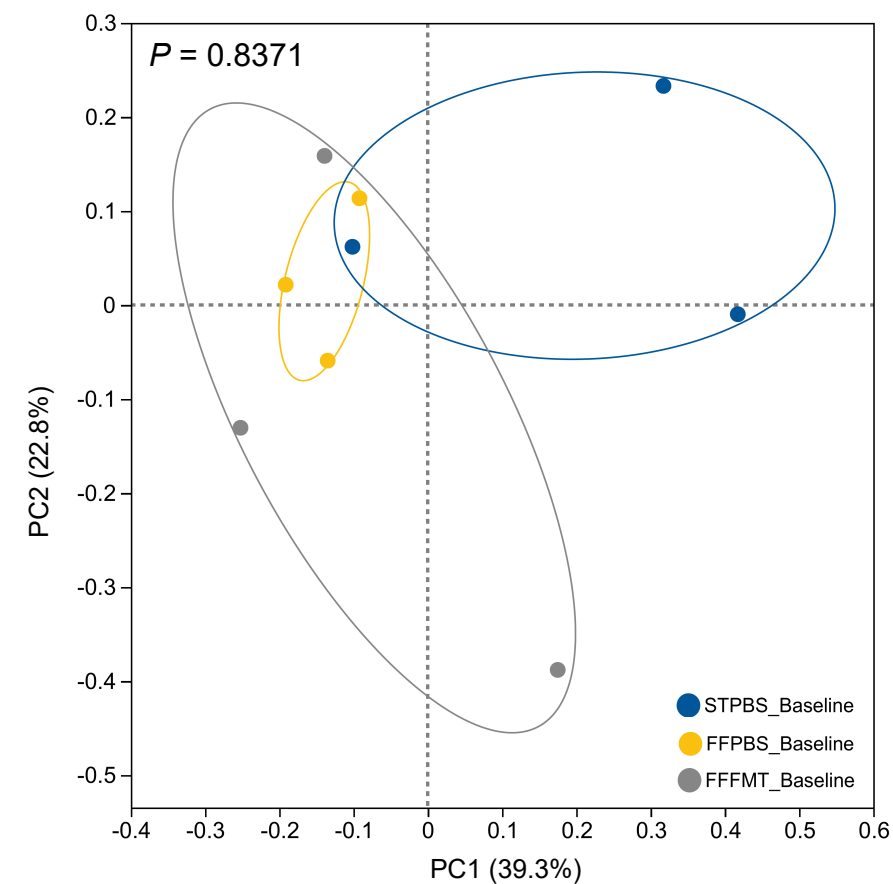**D**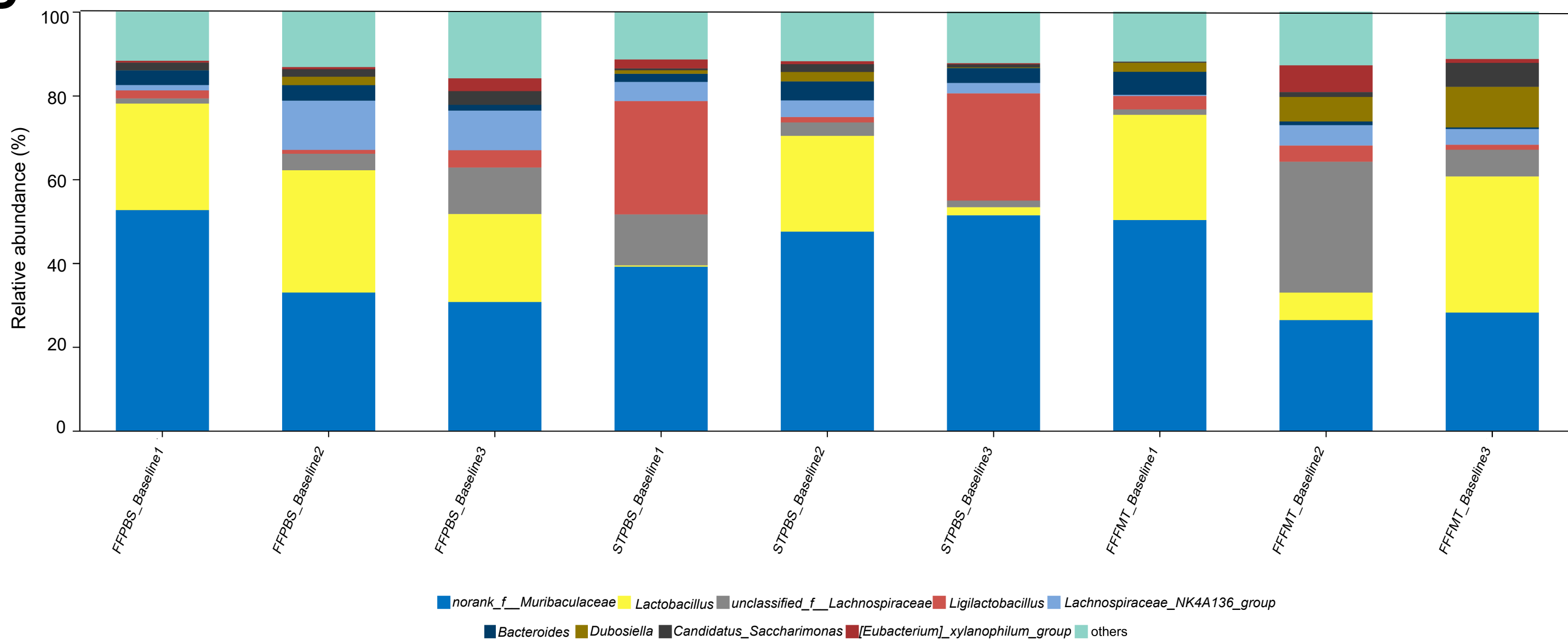

Supplement: Figure_S7_wraf069 [file figure_s7_wraf069.pdf]

**A**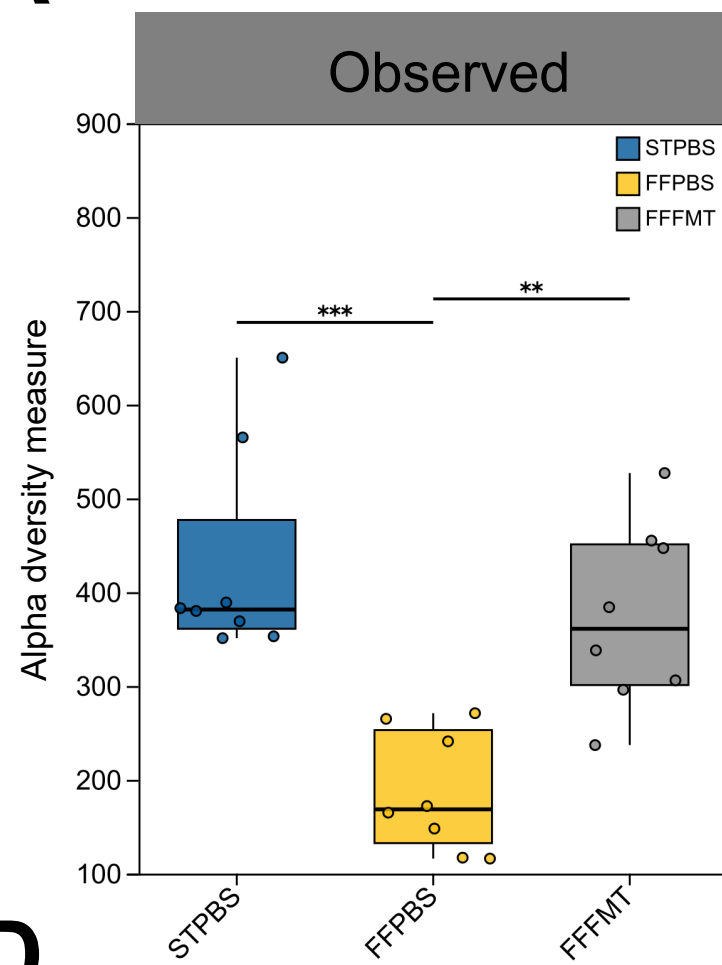**B**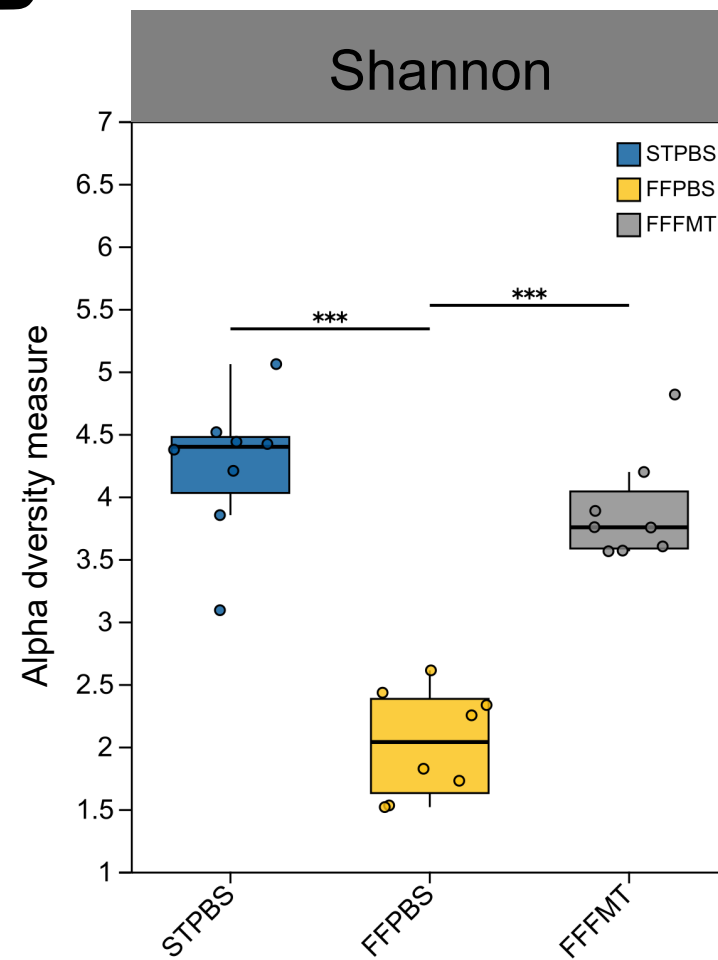**C**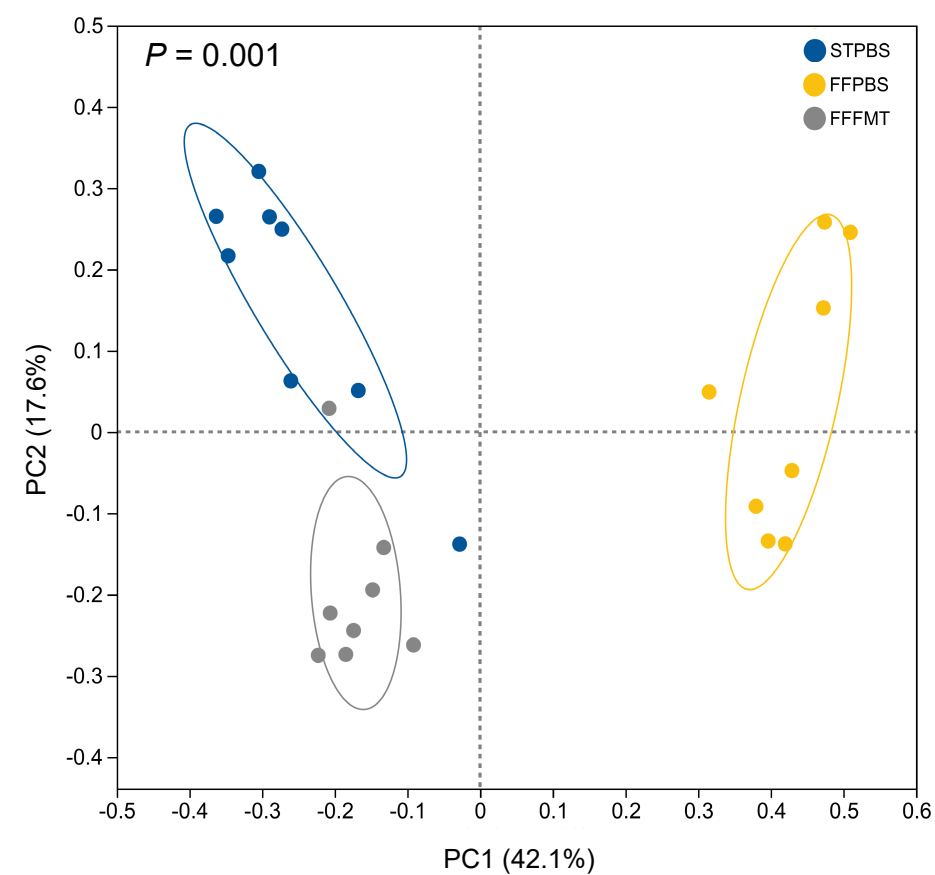**D**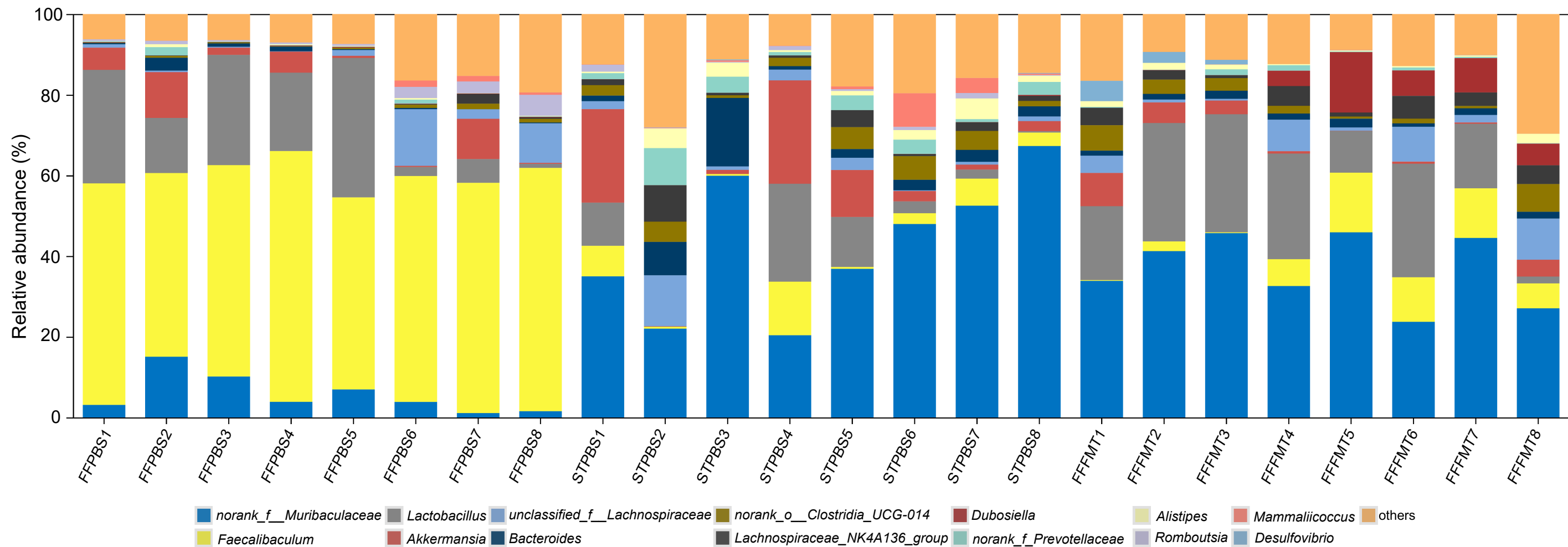

Supplement: Figure_S8_wraf069 [file figure_s8_wraf069.pdf]

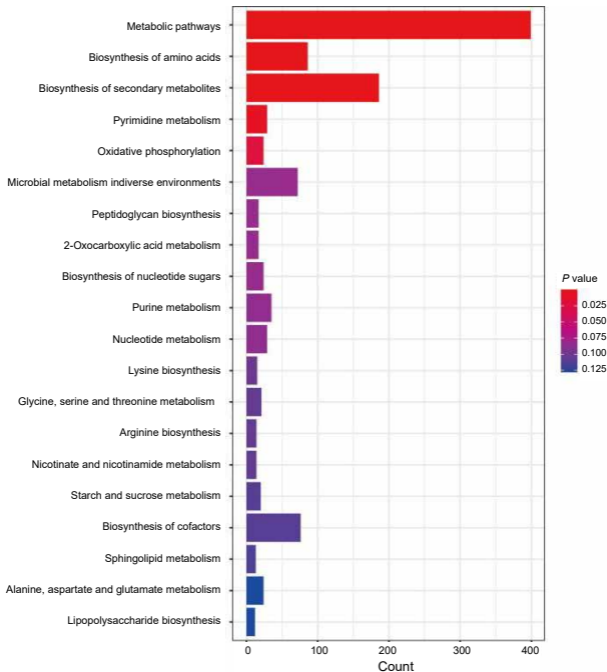

Supplement: Figure_S9_wraf069 [file figure_s9_wraf069.pdf]

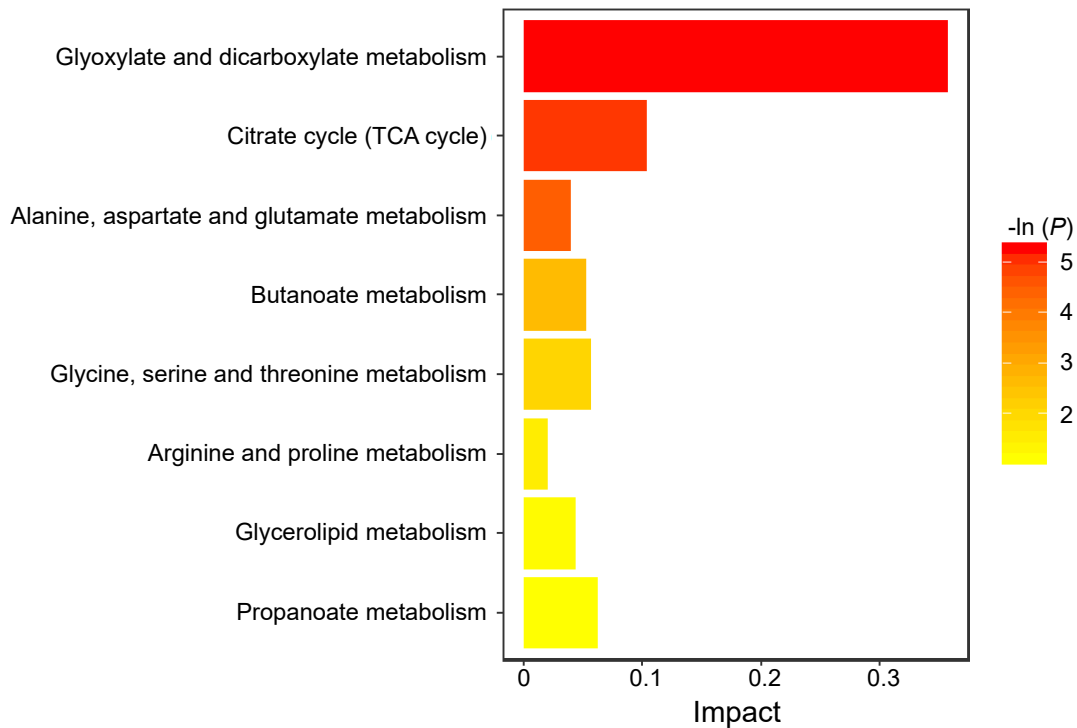

Supplement: Figure_S11_wraf069 [file figure_s11_wraf069.pdf]

**A**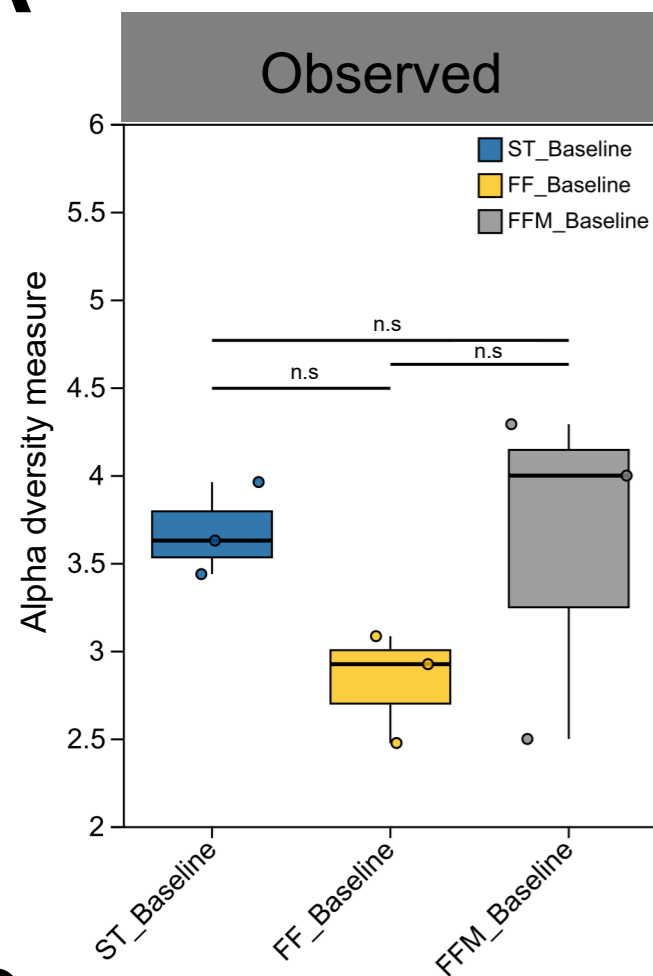**B**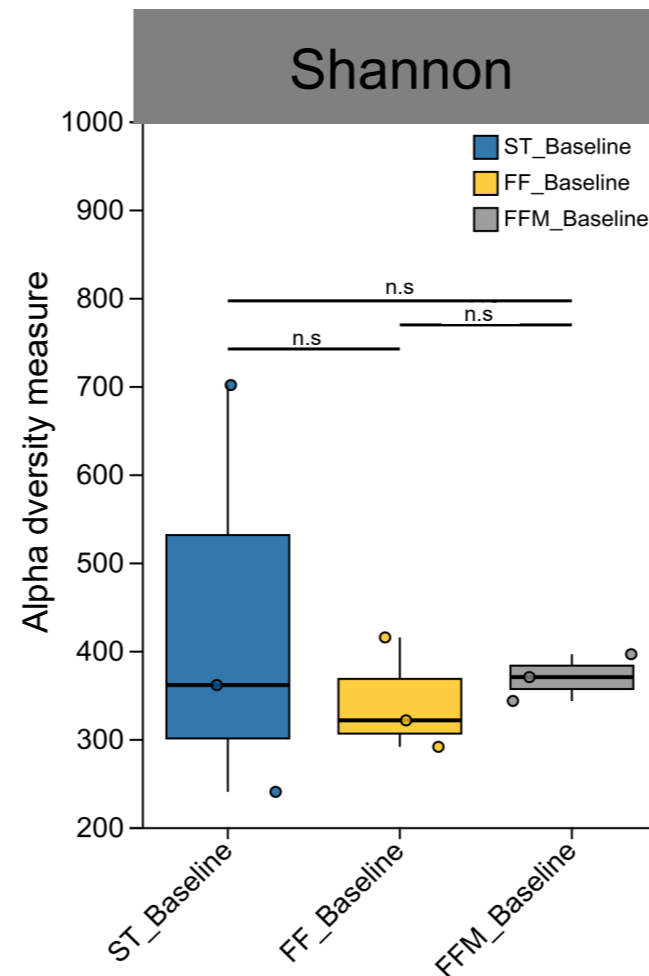**C**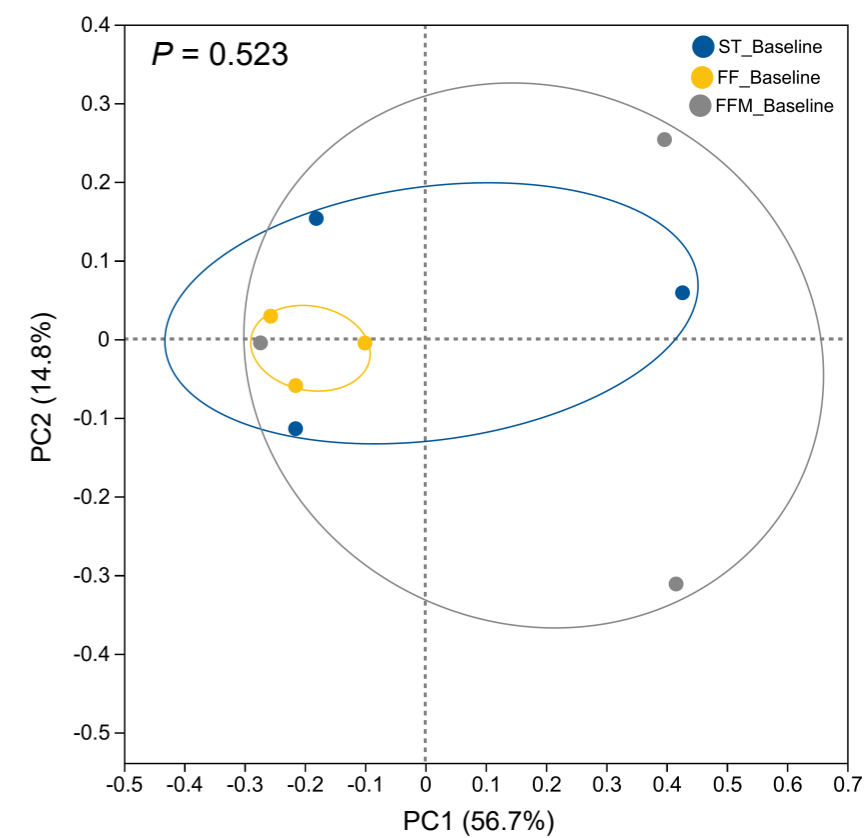**D**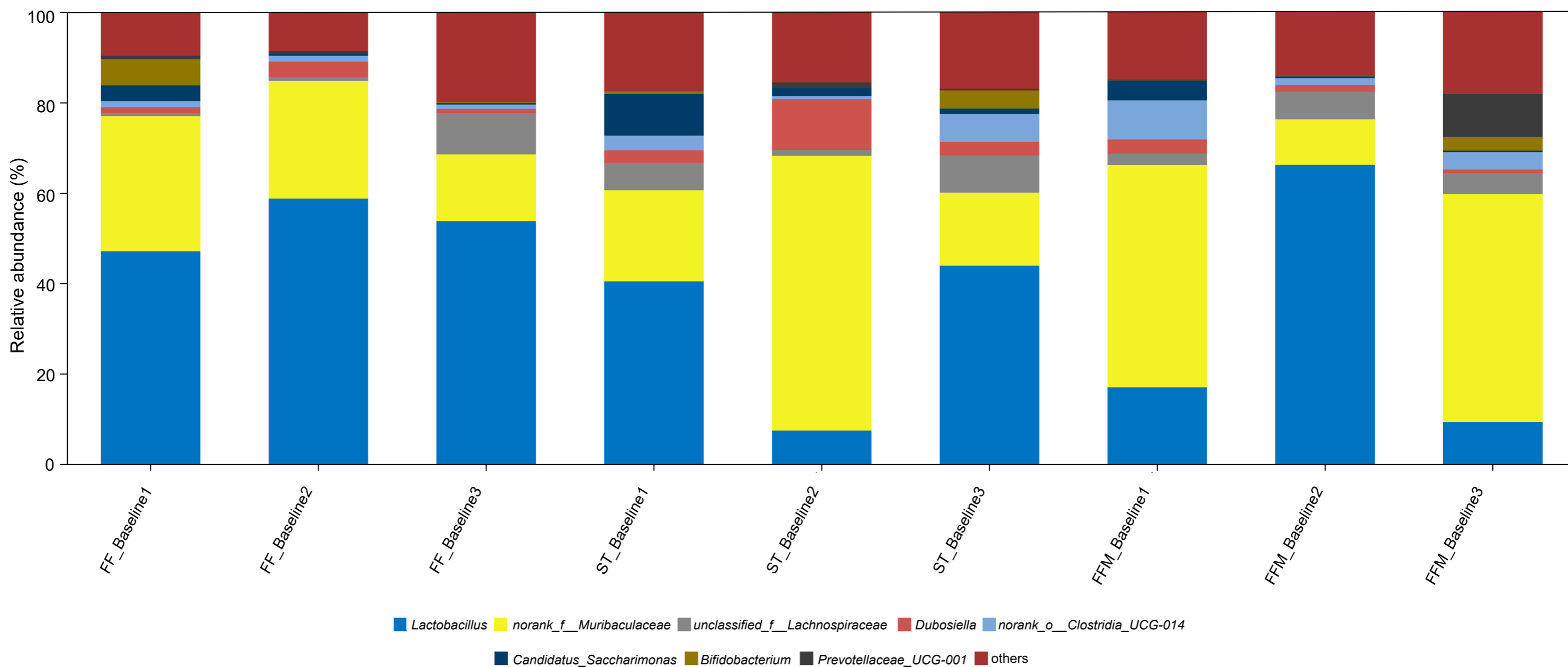

Supplement: Figure_S12_wraf069 [file figure_s12_wraf069.pdf]

**A**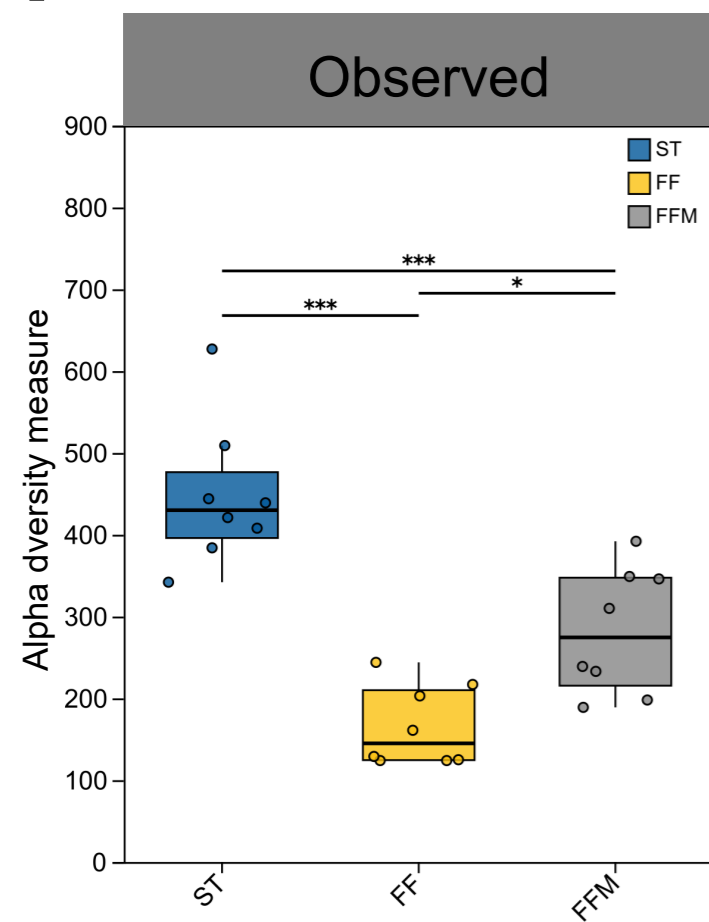**B**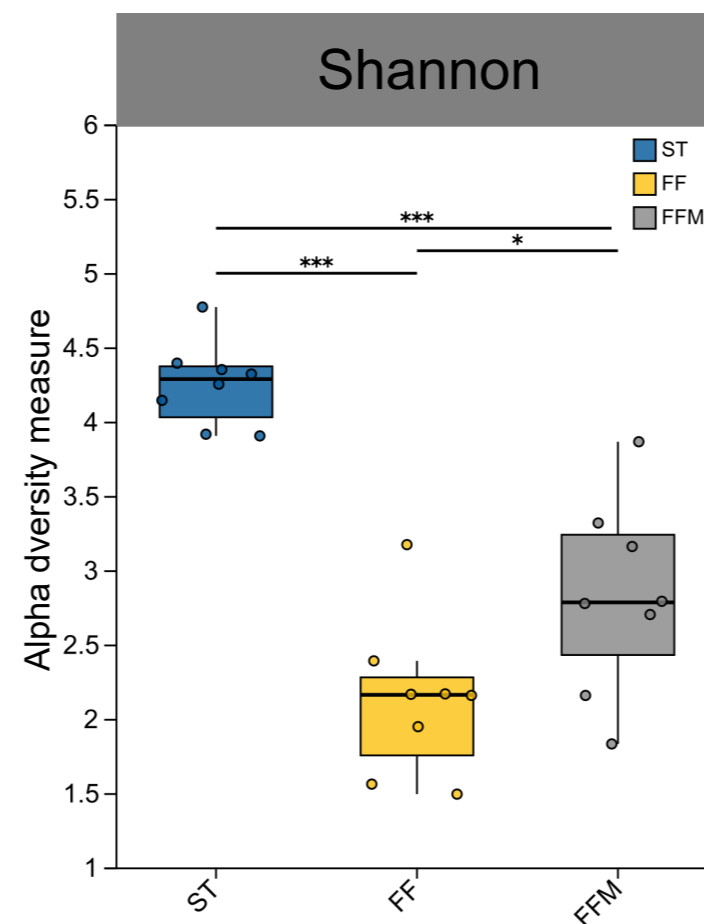**C**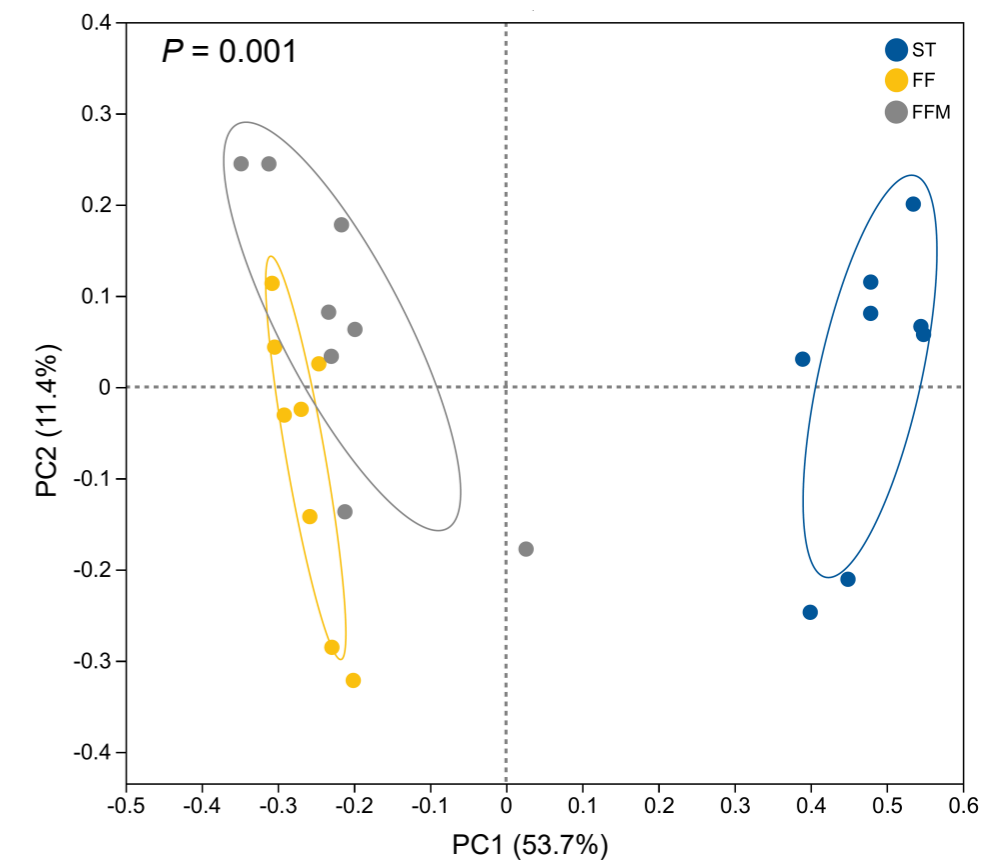**D**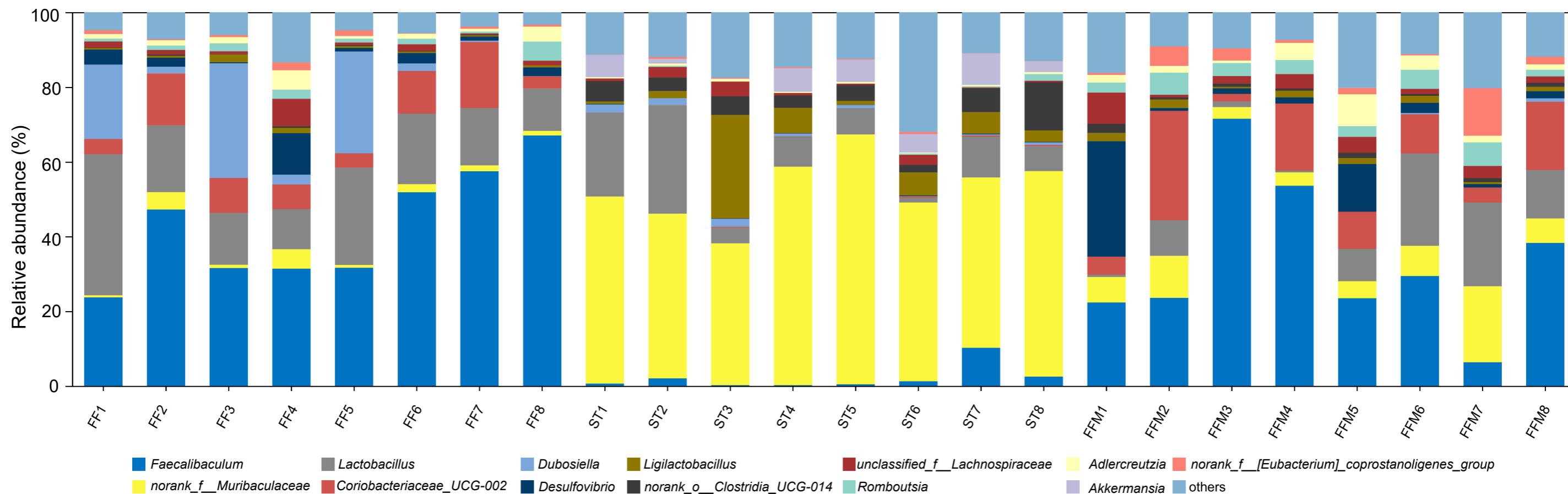

Supplement: Figure_S13_wraf069 [file figure_s13_wraf069.pdf]
