# Supplementary material for: Muribaculum intestinale restricts Salmonella Typhimurium colonization by converting succinate to propionate
Source: ISME J. 2025 Apr 18;19(1):wraf069. doi: 10.1093/ismejo/wraf069 (PMC12064562; doi:10.1093/ismejo/wraf069)
Supplement: supplementary_information_4_8_wraf069 [file supplementary_information_4_8_wraf069.docx]

**Supplementary Materials**

***Muribaculum intestinale* restricts *Salmonella* Typhimurium colonization by converting succinate to propionate**

Zhenyu Wang^1 #^, Shuaishuai Kang^1 #^, Zhenhua Wu^1^, Xiaoyi Liu^1^, Xiangyu Zhang^1^, Yujun Wu^1^, Yang Wen^1^, Xingjian Zhou^1^, Guolong Zhang^2^, Junjun Wang^1^, Dandan Han^1 *^

**Authors and Affiliations:**

^1^ State Key Laboratory of Animal Nutrition and Feeding, College of Animal Science and Technology, China Agricultural University, Beijing100193, China

^2^ Department of Animal and Food Sciences, Oklahoma State University, Stillwater, OK 74078, USA

#These two authors contributed equally.

***Address for reprint requests and other correspondence:** Dandan Han, State Key Laboratory of Animal Nutrition and Feeding, College of Animal Science and Technology, China Agricultural University, No. 2 West Yuanmingyuan Road, Beijing, China 100193 (e-mail: [handandan@cau.edu.cn](mailto:wangjj@cau.edu.cn)).

**Figure S1. Dietary fiber deprivation compromises colonization resistance against** ***S.* Typhimurium infection in mice.** Male C57BL/6J mice of 6-8 weeks were fed either a standard chow (ST) or a fiber-free (FF) diet for 7 or 28 days, followed by infection with *S.* Typhimurium and sample collection at different time points post-infection (pi) (*n* = 6-8). (A) Fecal *S.* Typhimurium loads in mice fed the ST or FF diet for 7 days at indicated times post-infection. (B) Fecal *S.* Typhimurium loads in mice fed the ST or FF diet for 28 days at indicated times post-infection. (C) *S.* Typhimurium loads in the liver, spleen, and mesenteric lymph node (MLN) of mice fed the ST or FF diet for 7 days at indicated times post-infection. (D) The *S*. Typhimurium loads in the liver, spleen, and mesenteric lymph nodes (MLN) of mice fed the ST or FF diet for 28 days at indicated times post-infection. ^*^ *P* < 0.05, n.s., not significan

**Figure S2. Baseline fecal microbiota compositions of mice prior to being fed either a ST or a FF diet for 7 or 28 days**. (A-B) Boxplots showing alpha-diversity of the two groups, expressed as observed species and Shannon index. (C) PCoA plots of the fecal microbiota based on Bray-Curtis metrics. (D) Individual fecal microbiota compositions in the two groups of mice.

**Figure S3. Fecal microbiota compositions of mice fed either a ST or a FF diet for 28 days prior to** ***S.* Typhimurium challenge**. (A-B) Boxplots showing alpha-diversity of the two groups, expressed as observed species and Shannon index. (C) PCoA plots of the fecal microbiota based on Bray-Curtis metrics. (D) Individual fecal microbiota compositions in the two groups of mice. ^**^ *P* < 0.01.

**Figure S4. Colonic microbiota compositions of mice fed either a ST or a FF diet at 4 days post-infection with *S.* Typhimurium.** (A) Individual colonic microbiota compositions at the phylum level. (B) Individual colonic microbiota compositions at the genus level.

**Figure S5. Reversion to standard diet restores colonization resistance against *S.* Typhimurium infection**. Mice were fed either a ST or a FF diet for 28 days, followed by an infection with *S*. Typhimurium and sample collection at 4 dpi (n = 10). A third group of mice (FFST14) were fed the FF diet for 28 days and switched back to the ST diet for another 14 days prior to *S*. Typhimurium infection. (A) Fecal *S.* Typhimurium loads among the three groups of mice at indicated times post-infection. (B) *S.* Typhimurium loads in the liver, spleen, and mesenteric lymph node (MLN) of mice among the three groups of mice at 4 days post-infection.

**Figure S6. Colonic microbial composition of mice restored to a ST after feeding a FF at 4 days post-infection with *S*. Typhimurium.** (A) Individual colonic microbiota compositions at the phylum level. (B) Individual colonic microbiota compositions at the family level.

**Figure S7. Baseline fecal microbiota compositions of mice used in the fecal microbiota transplantation (FMT) study. The colonic microbiota compositions were analyzed in three mice in each of the three groups before others were fed either a ST or a FF diet for subsequent FMT**. (A-B) Boxplots showing alpha-diversity of the three groups, expressed as observed species and Shannon index. (C) PCoA plots of the fecal microbiota based on Bray-Curtis metrics. (D) Individual fecal microbiota compositions among the three groups of mice. n.s., not significant.

**Figure S8. Fecal microbiota compositions of mice used in the fecal microbiota transplantation (FMT) study.** Mice were fed either a ST or a FF diet for 28 days, followed by daily gavage with PBS or fecal microbiota of ST-fed mice (FMT) daily for 14 days. Fecal microbiota was analyzed (n = 8) prior to *S*. Typhimurium challenge**.** (A-B) Boxplots showing alpha-diversity of the three groups, expressed as observed species and Shannon index. (C) PCoA plots of the fecal microbiota based on Bray-Curtis metrics. (D) Individual fecal microbiota compositions among the three groups of mice. ^**^ *P* < 0.01, ^***^ *P* < 0.001.

**Figure S9. Pathway enrichment analysis of the *Muribaculum intestinale* genome**. Top 20 KEGG pathways encoded in the *Muribaculum intestinale* genome are shown.

**Figure S10. *Muribaculum intestinale* genome encodes the biosynthetic metabolic pathway for converting succinate to propionate**.

**Figure S11. Enrichment analysis of differentially abundant metabolites in the feces of mice fed a fiber-free diet*.***

**Figure S12. Baseline fecal microbiota compositions of mice used in the *M. intestinale* administration study. The colonic microbiota compositions were analyzed in three mice in each of the three groups before others were fed either a ST or a FF diet for subsequent oral administration of *M. intestinale***. (A-B) Boxplots showing alpha-diversity of the three groups, expressed as observed species and Shannon index. (C) PCoA plots of the fecal microbiota based on Bray-Curtis metrics. (D) Individual fecal microbiota compositions among the three groups of mice. n.s., not significant.

**Figure S13. Fecal microbiota compositions of mice in the *M. intestinale* administration study.** Mice were fed either a ST or a FF diet (*n* = 10). On day 28, they were gavaged with PBS or 1 × 10^8^ CFU *M. intestinale* daily for 14 days, followed by colonic microbiota analysis**.** (A-B) Boxplots showing alpha-diversity of the three groups, expressed as observed species and Shannon index. (C) PCoA plots of the fecal microbiota based on Bray-Curtis metrics. (D) Individual fecal microbiota compositions among the three groups of mice. ^*^ *P* < 0.05, ^***^ *P* < 0.001.
