# Supplementary material for: Muribaculum intestinale restricts Salmonella Typhimurium colonization by converting succinate to propionate
Source: ISME J. 2025 Apr 18;19(1):wraf069. doi: 10.1093/ismejo/wraf069 (PMC12064562; doi:10.1093/ismejo/wraf069)
Supplement: Figure_S10_wraf069 [file figure_s10_wraf069.pdf]

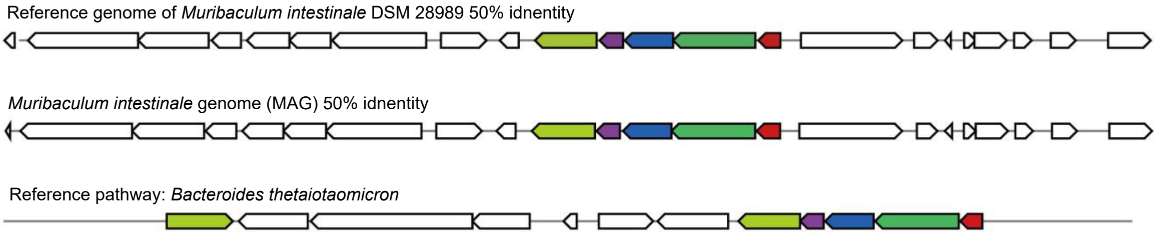
Reference genome of *Muribaculum intestinale* DSM 28989 50% idnentity
Muribaculum intestinale genome (MAG) 50% idnentity
Reference pathway: *Bacteroides thetaiotaomicron*
